# MircroRNA Let-7a-5p in Airway Smooth Muscle Cells is Most Responsive to High Stretch in Association With Cell Mechanics Modulation

**DOI:** 10.3389/fphys.2022.830406

**Published:** 2022-03-25

**Authors:** Kang Wen, Kai Ni, Jia Guo, Bing Bu, Lei Liu, Yan Pan, Jingjing Li, Mingzhi Luo, Linhong Deng

**Affiliations:** Changzhou Key Laboratory of Respiratory Medical Engineering, Institute of Biomedical Engineering and Health Sciences, Changzhou University, Changzhou, China

**Keywords:** ventilator-induced lung injury, lung compliance, airway smooth muscle cells, microRNA, let-7a-5p, biomechanics

## Abstract

**Objective:** High stretch (strain >10%) can alter the biomechanical behaviors of airway smooth muscle cells which may play important roles in diverse lung diseases such as asthma and ventilator-induced lung injury. However, the underlying modulation mechanisms for high stretch-induced mechanobiological responses in ASMCs are not fully understood. Here, we hypothesize that ASMCs respond to high stretch with increased expression of specific microRNAs (miRNAs) that may in turn modulate the biomechanical behaviors of the cells. Thus, this study aimed to identify the miRNA in cultured ASMCs that is most responsive to high stretch, and subsequently investigate in these cells whether the miRNA expression level is associated with the modulation of cell biomechanics.

**Methods:** MiRNAs related to inflammatory airway diseases were obtained via bioinformatics data mining, and then tested with cultured ASMCs for their expression variations in response to a cyclic high stretch (13% strain) simulating *in vivo* ventilator-imposed strain on airways. Subsequently, we transfected cultured ASMCs with mimics and inhibitors of the miRNA that is most responsive to the high stretch, followed by evaluation of the cells in terms of morphology, stiffness, traction force, and mRNA expression of cytoskeleton/focal adhesion-related molecules.

**Results:** 29 miRNAs were identified to be related to inflammatory airway diseases, among which let-7a-5p was the most responsive to high stretch. Transfection of cultured human ASMCs with let-7a-5p mimics or inhibitors led to an increase or decrease in aspect ratio, stiffness, traction force, migration, stress fiber distribution, mRNA expression of *α*-smooth muscle actin (SMA), myosin light chain kinase, some subfamily members of integrin and talin. Direct binding between let-7a-5p and ItgαV was also verified in classical model cell line by using dual-luciferase assays.

**Conclusion:** We demonstrated that high stretch indeed enhanced the expression of let-7a-5p in ASMCs, which in turn led to changes in the cells’ morphology and biomechanical behaviors together with modulation of molecules associated with cytoskeletal structure and focal adhesion. These findings suggest that let-7a-5p regulation is an alternative mechanism for high stretch-induced effect on mechanobiology of ASMCs, which may contribute to understanding the pathogenesis of high stretch-related lung diseases.

## Introduction

Human airways continually experience various extents of mechanical stretch *in vivo*, which plays essential roles in both physiological processes such as airway structural formation and functional maintaining, and pathological processes such as ventilator-induced lung injury (VILI) (Slutsky, 1999; Pidaparti and Koombua, 2011; Cox and Carson, 2012). In fact, VILI is a common risk in using mechanical ventilation (MV) to provide life support for acute respiratory distress syndrome (ARDS) patients such as the critically ill patients with coronavirus disease 2019 (COVID-19) because the patients under MV are widely reported to be subjected to high stretch (strain >10%) in the lungs (Sinclair et al., 2007; Phua and Govert, 2008; Ibrahim et al., 2016; Bagchi and Vidal Melo, 2018; Retamal et al., 2018; Pannone et al., 2021).

Considering the high mortality rate of patients under MV (e.g., 30–60% in COVID-19) (Grieco et al., 2020), it is very important to understand the role of mechanical stretch in the pathological mechanisms of VILI. Previous studies have proposed several pathways through which stretch might contribute to the pathogenesis and pharmacological intervention of VILI (Haitsma et al., 2000; Mendez et al., 2004; Uhlig and Uhlig, 2004; Vlahakis and Hubmayr, 2005; Oeckler and Hubmayr, 2008). However, none of these have led to really effective reduction of the mortality rate of ARDS patients under MV, which suggests a need for exploring alternative pathological mechanisms of VILI (Slutsky and Ranieri, 2013; Cabrera-Benitez et al., 2014; Fan et al., 2017; Villar et al., 2021). One area of interest is the role of stretch in changing biomechanical behaviors of airway smooth muscle cells (ASMCs) and the associated regulatory signaling via noncanonical molecular pathways such as microRNAs (miRNAs) (Farooqi et al., 2020). This is because that ASMCs are known as the major mechanosensitive cells in airways and have been reported to respond to stretch by changing contractile and secretory phenotypes in respiratory diseases such as asthma (Bartolák-Suki et al., 2014; Schwingshackl, 2016; Asano et al., 2018). Therefore, ASMCs may also play a crucial role in responding to high stretch during MV and mediate the pathogenesis of VILI.

Early studies have demonstrated that ASMCs respond to contractile agonists to decrease lung compliance (Mitzner et al., 1992). Since lung compliance refers to the ability of the lung to expand with changing pressure, changes in lung compliance reflect a pathological alteration in respiratory structure and function that affects the ventilation effect during MV (Shapiro and Bartlett, 1992; Katira, 2019). For example, COVID-19 patients under MV show reduced lung compliance, which is linearly related to impairment of pulmonary oxygenation (Grieco et al., 2020; Cronin et al., 2021; Tsumura et al., 2021). It has also been reported that patients under MV for over 6 days with decreased lung compliance are associated with an increased risk of death (Seeley et al., 2011). Thus, the decreased lung compliance may be a direct and easily measured marker for risk and progression of VILI (Cortes-Puentes et al., 2015; Nair et al., 2021). Although this decreased lung compliance is mainly determined by the loss of alveolar space, it is also partly affected by the biomechanics of ASMCs in small airways, and the latter is well known to be altered by stretch (Mauri et al., 2017; Guérin et al., 2022). However, the regulatory mechanism of stretch-induced mechanical response in ASMCs is still not fully elucidated although a multitude of molecular pathways have been identified to participate in this phenomenon.

Recently, emerging evidence implicates a critical role of miRNAs in modulating airway inflammation as novel signaling regulating molecules (Maneechotesuwan, 2019). For example, several miRNAs, including miR-23b, miR-24, miR-30b, miR-451, and the let-7 family are highly expressed in the lung (Lu et al., 2009). And miRNAs such as let-7a can regulate many mechanically associated molecules such as interleukin 13 (IL-13) and integrin (Itg) (Müller and Bosserhoff, 2008). However, it still remains unclear whether miRNAs are involved in response of ASMCs to high stretch and their underlying mechanisms, in particular the relationship between high stretch and the expression of miRNA and modulation of mechanical behaviors of ASMCs (Alipoor et al., 2016).

To address this question, we first conducted bioinformatics data mining to identify miRNAs that are associated with airway inflammation. Then we tested ASMCs in culture for response to high stretch (>10% strain), in terms of expression of the miRNAs. As a result, we identified that among the inflammation-associated miRNAs, let-7a-5p was most responsive to high stretch stimulation. Then, we demonstrated that the expression of let-7a-5p indeed impacted ASMCs in terms of morphology and mechanical behaviors. These findings indicate that high stretch can be a potent stimulus to induce expression of stretch-responsive miRNAs such as let-7a-5p, which in turn can modulate the structure and function of ASMCs. Thus it might be a useful strategy to target miRNAs that are responsive to stretch for preventing and/or treating high stretch-related lung diseases.

## Materials and Methods

### Materials

Transferrin (#T8158) and insulin (#91077C) were purchased from Sigma-Aldrich (St. Louis, MO, USA). Collagen type I (#08–115) was purchased from Advanced BioMatrix (#5279, Poway, CA, USA). Dulbecco’s modified Eagle’s medium (#11885092, DMEM), fetal bovine serum (FBS, #16000-044), penicillin-streptomycin (#15140122) and trypsin (#25200056) were purchased from Thermo Fisher Scientific (Waltham, MA, USA). Cell culture flasks (#3073) and plates (#CLS3506) were purchased from Corning Incorporated (Corning, NY, USA). All other reagents were purchased from Thermo Fisher Scientific unless noted otherwise.

### Identification of High Stretch Responsive miRNAs in ASMCs

In order to identify high stretch responsive miRNAs in ASMCs, we first carried out bioinformatics analysis by using Human MicroRNA disease database (HMDD v3.0, http://www.cuilab.cn/hmdd) to collect all miRNAs that are reported to be associated with airway diseases including asthma, chronic obstructive pulmonary disease (COPD), and lung injury. Then we examined these collected miRNAs in the PubMed database up to 2020 for their annotations, and those with annotations containing inflammation signaling and reference review were considered to be associated with airway inflammation. Subsequently, we evaluated the expressions of all the inflammation-associated miRNAs in cultured ASMCs in response to high stretch treatment, which ultimately identified high stretch responsive miRNAs in ASMCs.

### Culture of ASMCs With/Out High Stretch Treatment

Two primary human ASMCs cell strains were purchased from ScienCell Research Laboratories, Inc. (#3400, Carlsbad, CA) and BeNa Culture Collection (#BNCC339826, Beijing, China), respectively. The former cell strain was maintained in smooth muscle cell medium (SMCM, #1101) supplemented with 1% smooth muscle cell growth supplement (SMCGS, #1152), and the latter cell strain was maintained in the DMEM basal cell culture medium. All cell culture media were supplemented with 10% FBS, 2 mM l-glutamine, 100 units/mL penicillin, and μg/ml streptomycin. Cells were cultured in an incubator containing 5% CO_2_ humidified at 37 C. Prior to the experiment, exponential proliferating ASMCs (2 × 10^4^ cells/cm^2^) at passage 3-10 were plated on type I collagen-coated Bioflex 6-well plates and were serum-deprived for 24 h. Since the stretch of airway walls in normal lungs during tidal respiration is estimated to be about 5% and the stretch in ARDS with MV can be twice as large (Deng et al., 2004; Deng et al., 2009; Bagchi and Vidal Melo, 2018; Retamal et al., 2018), we exposed ASMCs grown on Bioflex plates to a 13% cyclic strain at 0.5 Hz for 48 h (a sinusoidal wave, 1 s of deformation alternating with 1 s of relaxation, Flexcell 5,000, FlexCell International, Hillsborough, NC, USA) to simulate the high stretch on the cells in ARDS during MV. For ASMCs without high stretch treatment, the cells were grown in Bioflex plates and maintained in static conditions for 48 h. The cells with or without high stretch treatment were subsequently used as experiment or control groups, respectively.

### Isolation and Quantification of miRNA and mRNA From ASMCs

Total RNA was purified using TRI Reagent RNA Isolation Reagent (#T9424, Sigma-Aldrich, St. Louis, MO, USA), and the extracted RNA was quantified on Nanodrop 2000 spectrophotometer (Thermo Scientific, Willmington, DE, USA). For miRNA quantification, miRNA 1st Strand cDNA Synthesis Kit (by stem-loop) (#MR-101, Vazyme Biotech, Nanjing, China) was used for reverse transcription, real-time PCR was performed with miRNA universal SYBR qPCR Master Mix (#MQ-101, Vazyme Biotech, Nanjing, China) using a StepOne real-time PCR system (Applied Biosystems, Carlsbad, CA, USA) at 95 C for 5 min, followed by 40 cycles of 95 C for 10 s, 55 C for 30 s and 72 C for 30 s. The primers for the target genes were synthesized by General Biosystems (Anhui, China). Each reaction used 1 μl of cDNA, 0.4 μl of each primer, 0.4 μl of MQ Primer R (10 μM), 10 μl of 2 x miRNA universal SYBR qPCR Master Mix, and Ultrapure™ Nuclease-Free Water (Qiagen, Hilden, Germany) to reach a total reaction volume of 20 μl. Each sample was run in triplicate. The Rnu6 (U6, General Biosystems, Anhui, China) was chosen as an endogenous reference for miRNA normalization. The primer sequences are shown in Supplementary Table S1.

For mRNA quantification, 500 ng total RNA was used to generate 1st strand cDNA using the Revert Aid First Strand cDNA Synthesis Kit (#K1622, Thermo Scientific, MA, USA). The associated primers used were shown in Supplementary Table S2 and purchased from General Biosystems (Anhui, China). Real-time PCR was performed with PowerUp SYBR Green Master Mix (#A25742, Applied Biosystems, CA) using the StepOne real-time PCR system (Applied Biosystems) at 50 C for 2 min, 95 C for 2 min, followed by 40 cycles of 95 C for 15 s, 55 C for 15 s and 72 C for 60 s. The reaction system contains 1 µl of cDNA in a 10 µl reaction according to the manufacturer’s instructions in triplicate.

Calibration and normalization were done using the 2^−^^∆∆CT^ method, where ∆C_T_ = C_T_ (target gene) - C_T_ (reference gene) and ∆∆C_T_ = ∆C_T_ (experiment groups) - ∆C_T_ (control groups). Fold changes in miRNA or mRNA expression of different genes were calculated as the ratio of experiment groups to the control groups from the resulting 2^−∆∆CT^ values from three independent experiments.

### Transfection of Let-7a-5p Mimics or Inhibitors Into ASMCs

The mature sequences of let-7a-5p mimics (5′-UGA​GGU​AGU​AGG​UUG​UAU​AGU​U-3), inhibitors (5′-AAC​UAU​ACA​ACC​UAC​UAC​CUC​A-3′), and their related mimics controls, (5′-UCA​CAA​CCU​CCU​AGA​AAG​AGU​AGA-3′), inhibitor controls (5′-CUACUCUUUCUAGG

AGGUUGUGA-3′) were obtained from General Biosystems (Anhui, China). ASMCs were seeded in plates, and once cells reached 50–80% confluence, they were transfected with either let-7a-5p mimics or inhibitors and corresponding scramble controls (50 nM) by Lipofectamine 3,000 reagent (#L3000015, Thermo Fisher Scientific, Waltham, MA, USA) following the manufacturer’s instructions. After 12 h incubation, the FBS-free Opti-MEM medium was replaced with DMEM medium without FBS. The cells were further incubated for 48 h before being used in the subsequent experiment.

### Assessment of Viability of ASMCs

The procedure for the cell counting Kit-8 (CCK-8) assay kit (#C0037, Beyotime Biotechnology, Shanghai, China) was as described previously (Jin et al., 2021). Cells were plated in 96-well plates at a density of 1×10^4^ cells/well. After overnight cultivation, the cells were transfected, and then 10 μl of CCK-8 solution was added to the cells for 4 h at 37°C. Absorbance was acquired by using an automatic microplate reader at 450 nm (Infinite F50, Tecan, Männedorf, Switzerland).

### Assessment of Morphological Changes and Stress Fiber Distribution of ASMCs

To observe the changes of cellular morphology of ASMCs, low density of ASMCs (0.5 × 10^5^ cells/cm^2^) were inoculated in 6-well plate (Corning, NY, USA) for 24 h and then were transiently transfected with let-7a-5p mimics or inhibitors for 24 h. Then cells were observed and recorded at 0, 6, 12, 18 and 24 h by using a Cell Observer System (Zeiss, Germany) equipped with a CO_2_ and temperature control chamber. The cellular length and width were measured manually in ImageJ software and cellular aspect was defined by the ratio of the cellular length on the width of single cells. For each group, 30 cells were observed and analyzed.

For immunofluorescent staining, ASMCs were transiently transfected with let-7a-5p mimics or inhibitors and related negative controls for 48 h, then cell fixation was performed in 3.7% paraformaldehyde for 15 min at room temperature (RT), then the cells were permeabilized with 0.5% Tween-X-100 for 15 min at RT. Nonspecific binding of the antibodies was blocked by incubating the samples in phosphate buffered saline (PBS) containing 1% bovine serum albumin (BSA) for 1 h at RT. F-actin (red) was stained with fluorescent red phalloidin for 20 min and nuclei (blue) were stained with DAPI for 3 min in the dark. Between all staining steps, cells were washed 3 times with PBS. Stained cells were visualized by using laser scanning confocal microscopy at ×40 objective (LSM710, Zeiss, Germany). Acquisition parameters were kept constant during the experiments. Fluorescent images were processed with ImageJ (NIH, USA).

### Assessment of Cell Stiffness of ASMCs

Cell stiffness of cultured ASMCs was measured by optical magnetic twisting cytometry (OMTC) as described previously (Fabry et al., 2001; Smith et al., 2003; Deng et al., 2006; Coughlin et al., 2012; Luo et al., 2019). In brief, ASMCs cultured on type I collagen-coated dishes were incubated in serum-free culture media containing 5 ng/ml insulin and 5 ng/ml transferrin (IT medium) for 24 h. Then the cells were incubated with Arg-Gly-Asp-coated ferrimagnetic microbeads (4.5 μm) for 30 min and washed with DMEM to remove unbound beads. Then the microbeads were magnetized horizontally with a brief 1,000-G pulse followed by continuous twisting with a 20-G at 100 Hz vertically aligned sinusoidal magnetic field. The twisting magnetic field caused a rotation and a pivoting displacement of the bead. As the bead moved, the cell developed internal stresses to resist the bead motion. Thus, the lateral bead displacements in response to the resulting oscillatory magnetic torque were detected optically (in spatial resolution of 5 nm), and the ratio of specific torque to bead displacements was computed and expressed as the cell stiffness in units of Pascals (Pa) per nanometer.

### Assessment of Traction Force Generated by ASMCs

Cell traction force was measured by using Fourier transformation traction force microscopy as described previously (Dembo and Wang, 1999; Tolić-Nørrelykke et al., 2002; Wang et al., 2002; Wang et al., 2019a). Briefly, the confluence ASMCs were transiently transfected with let-7a-5p mimics or inhibitors and related negative controls for 24 h, and then seeded onto polyacrylamide gel embedded with fluorescent microbeads (0.2 μm, Molecular probes, Eugene, OR, USA) coated with type I collagen (0.1 mg/ml) in serum-free medium (IT medium) for 24 h (5,000 cells/dish), followed by microscopic imaging. The field of view for microscopic imaging was selected with a single cell; then cell shape and the fluorescent microbeads were then imaged by phase-contrast and fluorescence microscopy, respectively. Then NaOH was added to cell cultures and the cell-free bead positions were recorded as a reference point (traction-free) for bead displacement. Thus, the cell traction force was calculated by converting the displacement of microbeads (i.e., substrate deformation) to the force exerted by the cells on the substrate using proprietary Matlab software (MathWorks Corp., Natick, MA, USA) according to the images of the cells (phase contrast) and the microbeads (fluorescence) obtained before and after the NaOH treatment.

### Assessment of Migration of ASMCs

The horizontal cell migration was measured by using a modified scratch-wound healing method as previously described (Rodriguez et al., 2005). Briefly, a high density of cells (2 × 10^5^ cells/cm^2^) were inoculated into 6-well plates (Corning) and reached confluent monolayer, and then were transiently transfected with let-7a-5p mimics or inhibitors. An experimental wound was made using a sterile micropipette tip, then the cells were washed 3 times with sterile PBS. Wound areas were observed and recorded at 0, 6, 12, 18, and 24 h by using a Cell Observer System (Zeiss, Germany) equipped with a CO_2_ and temperature control chamber. The experimental wound area was quantified manually using “Area measurement” in ImageJ software and normalized to the wound area at the start of the experiment, and the migration distance was defined by the ratio of the wound healing area on the length of the wound area.

The vertical cell migration was measured by using 6-well transwell chambers that were separated as upper and lower chambers by filter membrane with 8 μm pores (#07-200-169, Corning). ASMCs were transiently transfected with let-7a-5p mimics or inhibitor and related negative controls for 36 h, then plated in the upper chamber (1 × 10^5^ cells/well), replaced with serum-free medium after 12 h, while the lower chamber was filled with 2 ml complete medium. After 24 h, cells were fixed with 4% paraformaldehyde (#30525-89-4, Electron Microscopy Sciences, Hatfield, PA). The non-invasive cells on the upper chamber were removed with cotton swabs, and the invaded cells in the lower chamber were stained with 0.1% crystal violet (#C6158, Sigma) for 10 min at RT, before being examined and imaged by light microscopy at ×10 objective (Olympus B×60, Olympus Corporation, Tokyo, Japan). Then the number of stained cells was counted using ImageJ software. Results were based on the analysis of 10 random fields per transwell in each condition and each experiment was repeated three times.

### Bioinformatics Analysis of Let-7a-5p Target Genes

Let-7a-5p was used as a keyword in miRmap (https://mirmap.ezlab.org/) database to search for target genes. The identified target genes were then used to perform Gene Ontology (GO) annotation and Kyoto Encyclopedia of Genes and Genomes (KEGG) pathway enrichment analyses by utilizing the database for Annotation, Visualization, and Integrated Discovery (DAVID, http://david.abcc.Ncifcrf.gov/) online software. The GO analysis included three categories: red for biological processes (BP), green for cellular components (CC), and blue for molecular functions (MF), then GO annotation and KEGG pathway enrichment analysis were plotted with GraphPad Prism 9.0 (Graph Pad Software, La Jolla, CA).

### Dual-Luciferase Reporter Assays in HEK293T Cells

The corresponding DNA sequence of the 3′-UTR of the integrin alpha V mRNA (4,255–4,356 bp, GGC​AAC​TCA​CTG​ATT​TAC​TTC​TAG​CAA​TAG​CAT​GAT​GTT​ACA​GGA​ATA​TTA​CC

TCT​GTT​TAA​GCA​AGG​TAA​TGT​GTA​AAA​TCA​GTC​TCG​GCT​GTC​AGA​ATA​A, target site for let-7a-5p: TACCTC) was cloned into the psiCHECK-2 vector (#VT1400, YouBio) downstream of the Renilla luciferase coding sequence via synthetic oligonucleotides ligation. In this assay, HEK293T cells (#BNCC353535, BNCC, Beijing, China, which are classical engineering cells used for dual-luciferase reporter assays since they have high transfection efficiency and less endogenous interferences) were plated at a density of 3 × 10^3^ cells per well in a 96-well plate and transiently transfected with 100 ng of the psiCHECK-2 vector or 100 ng of psiCHECK-2-ITGAV-3′-UTR. In addition, 50 nM of Let-7a-5p mimic or inhibitor (General Biosystems, Anhui, China) was also used in the transfection reactions processed with the Lipofectamine 3,000 transfection reagent (#L3000015, Thermo Fisher Scientific, USA). At 48 h post-transfection, the activities of luciferase were determined using the Dual luciferase Reporter Gene Assay Kit (#RG088S, Beyotime Biotechnology, Shanghai, China) according to the manufacturer’s instructions. The Firefly luciferase activity was normalized in each sample to account for differences in transfection efficiency. All assays were performed in triplicate.

### Statistical Analysis

Statistical analysis was performed by using GraphPad Prism 9.0 (Graph Pad Software, La Jolla, CA). Data were analyzed with a distribution test using Origin 2018 (OriginLab Corporation, Northampton, MA, USA). OMTC original data followed LogNormal distribution and thus were transformed by taking the natural log of all original data before analysis. Other data followed Normal distribution. Therefore, data were reported as means ± S.E.M, and n represents the number of samples. One-way analysis of variance (ANOVA) followed by Post ad-Hoc student’s t-test was carried out for multiple comparisons using Origin 2018. The significance of the mean comparisons is represented by asterisks (*p < 0.05; **p < 0.01).

## Results

### Let-7a-5p is the Most Responsive miRNA to High Stretch in ASMCs

Bioinformatics analysis using Human MicroRNA disease database (HMDD v3.0, http://www.cuilab.cn/hmdd) collected 32,281 disease-associated miRNA entries, including 1,102 miRNA genes, 850 diseases from 17,412 papers (Huang et al., 2019), among which 141 were identified to be associated with airway diseases including asthma, COPD and lung injury. Subsequently, we identified 29 out of the 141 miRNAs were involved in airway inflammation, resulting from analysis of the miRNAs for their annotations and relevant references in HMDD v3.0 and PubMed database up to 2020 (Supplementary Table S3).

**FIGURE 1:**
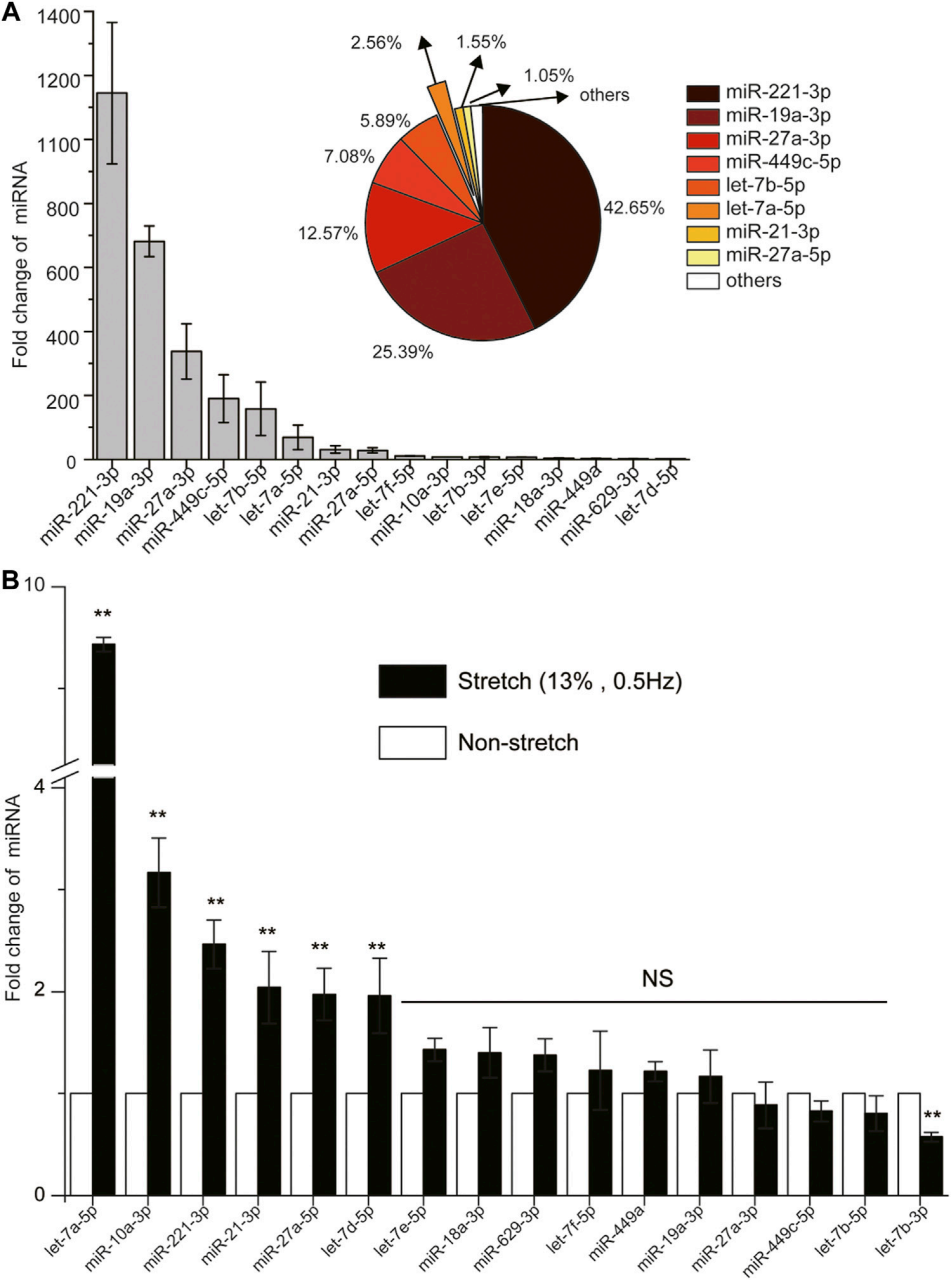
The expression of 16 miRNAs (cycle threshold, Ct value below 36) with/without high stretch in ASMCs. **(A)** The fold change of 16 miRNAs without high stretch. The pie chart in the top right insert shows the percentage distribution of the top eight highly expressed miRNAs of over 1% in ASMCs. **(B)** The fold change of 16 miRNAs in ASMCs with high stretch (13% strain, 0.5 Hz). All data are presented as means ± S.E.M. (n = 3). *p < 0.05, **p < 0.01, NS *p* > 0.05.

By using one primary human ASMCs cell strain from ScienCell that we have described (Wang et al., 2019b; Wang et al., 2019a), we found only 16 miRNAs to have cycle threshold (C_T_) value below 36 in ASMCs, indicating these 16 miRNAs to be more abundant and physiologically significant. The expression abundance of each miRNA in this group, measured as fold change of expression relative to that of U6 (reference gene) in the cells, ranked in the order of miR-221-3p > miR-19a-3p > miR-27a-3p > miR-449c-5p > let-7b-5p > let-7a-5p > miR-21-3p > miR-27a-5p > let-7f-5p > miR-10a-3p > let-7b-3p > let-7e-5p > miR-18a-3p > miR-449a > miR-629-3p > let-7d-5p (Figure 1A), and the eighth ranked miRNA already accounted for over 1% of the total expression of all these miRNAs (Pie chart in Figure 1A).

Then we evaluated the expression change of these 16 miRNAs in the cultured ASMCs from ScienCell after exposure to high stretch (13% strain) (Deng et al., 2004; Deng et al., 2009; Bagchi and Vidal Melo, 2018; Retamal et al., 2018). We found that among the 16 miRNAs, five were up-regulated in expression by more than 2-fold in response to high stretch, which ranked in the order of let-7a-5p > miR-10a-3p > miR-221-3p > miR-21-3p > miR-27a-5p > let-7days-5p (Figure 1B). Since let-7a-5p turned out to be the most responsive to high stretch, we assumed that let-7a-5p was most likely to be involved in the pathobiological process of VILI through modulation of biomechanics of ASMCs. Therefore, we focused on let-7a-5p in the following experiments for its effects on biomechanical behaviors of ASMCs.

### Let-7a-5p Modulates the Morphology and Stress Fiber Distribution of ASMCs

To explore the effects of let-7a-5p on ASMCs, let-7a-5p mimics or inhibitors were transiently transfected into ASMCs to either up or down-regulate the expression of let-7a-5p in the cells as compared to their counterparts transfected with scramble genes. The efficiency of transfection was then measured by miRNA qRT-PCR. When the ASMCs from ScienCell were transfected with let-7a-5p mimics or inhibitors, cells exhibited significant up or down-regulation of let-7a-5p expression by ∼800 fold or ∼13% (Supplementary Figure S1A left), but the cells transfected with let-7a-5p mimics exhibited significantly decreased viability as compared with controls (Supplementary Figure S1B left). Considering the unphysiological up-regulation of let-7a-5p and the impaired viability of ASMCs from ScienCell due to let-7a-5p mimics transfection, we ceased to use this cell strain in experiments for cells to be transfected with let-7a-5p mimics or inhibitors. Instead, we used the cell strain from BeNa Culture Collection. When transfected with let-7a-5p mimics or inhibitors, the cells from BeNa exhibited up or down-regulation of let-7a-5p by ∼4.5 fold or ∼34.5% (Supplementary Figure S1A right), while the cell viability remained unchanged as compared to the controls (Supplementary Figure S1B right). Furthermore, the cells from BeNa responded to high stretch with ∼8 fold increase of let-7a-5p expression, which was similar as the cells from ScienCell (Supplementary Figure S2). These data confirmed that in ASMCs from BeNa the high stretch and let-7a-5p transfection induced let-7a-5p up-regulation in the same order of magnitude, while not impairing the viability of the cells in culture. Therefore, we used ASMCs from BeNa in all the following experiments.

**FIGURE 2:**
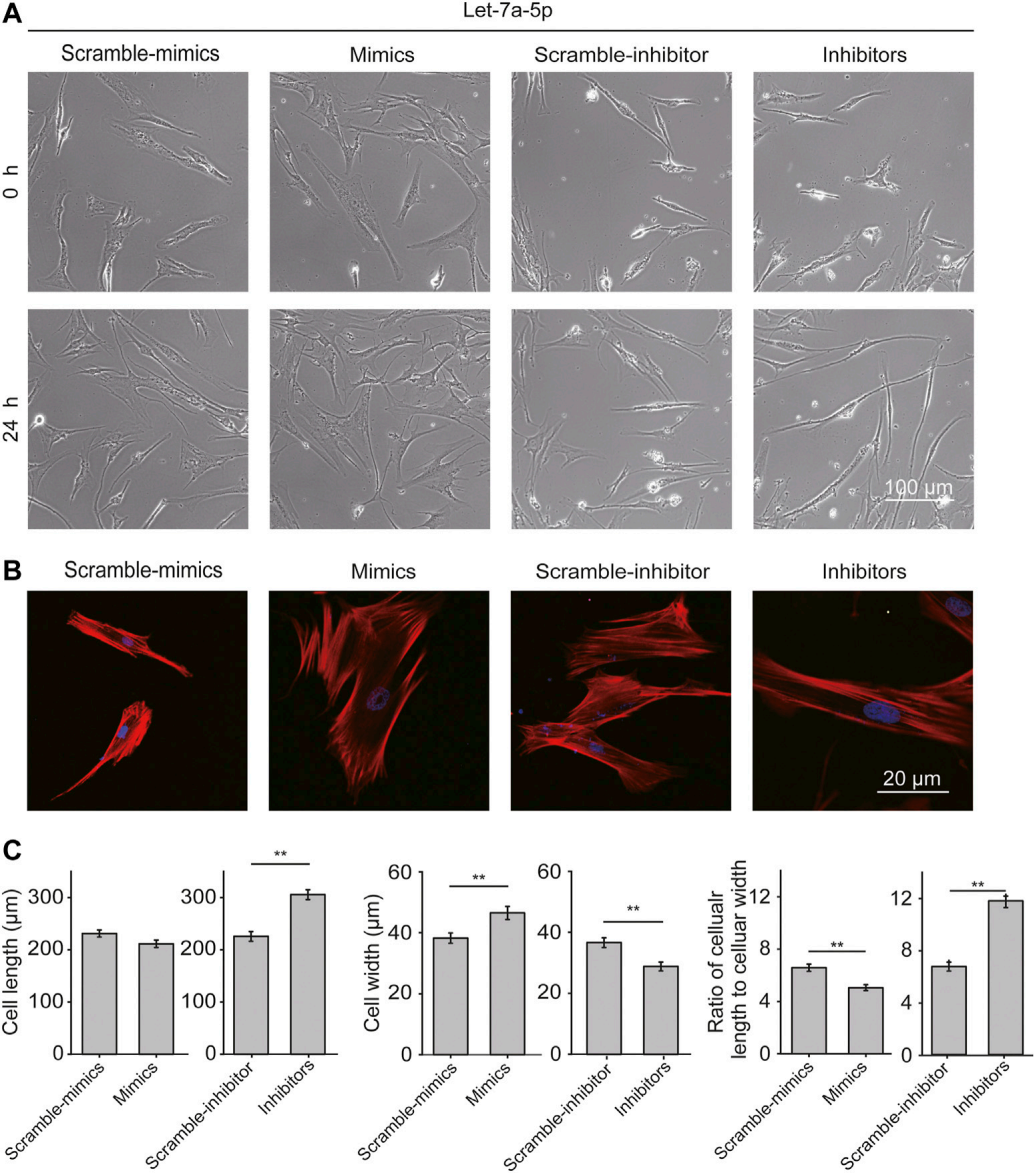
Let-7a-5p modulates cell morphology and stress fiber distribution. **(A)** The representative images of ASMCs transfected with let-7a-5p mimics, inhibitors, and related scramble controls. Bar = 100 µm. **(B)** The representative images of stress fiber in ASMCs. Stress fibers of ASMCs were stained with TRITC-Phalloidin and DAPI, respectively, in red and blue colors. Images were taken by confocal microscope (63X). Bar = 20 µm. **(C)** The quantitative data of cell morphology of ASMCs treated with let-7a-5p mimics, inhibitors, and related scramble controls. The cellular length and width were measured by ImageJ. Cell aspect ratio was defined as the ratio of cellular length to cellular width (*p < 0.05, **p < 0.01, n = 20).

ASMCs transfected with let-7a-5p mimics or inhibitors were observed for 24 h with time-lapse live-cell imaging. As shown in Figure 2A, ASMCs appeared in classical fibroblast-like shapes in all conditions. Interestingly, down-regulation of let-7a-5p turned most ASMCs into long worm-like shapes, resembling the freshly dissociated ASMCs that are considered more relevant to the morphology of ASMCs *in vivo* (Deng et al., 2006). Since cell morphology is determined by cytoskeleton especially the stress fibers, the effect of let-7a-5p on the distribution of stress fibers in ASMCs was evaluated by immunofluorescence analysis. We found that let-7a-5p down-regulation seemed to significantly increase the stress fibers length compared to the control groups (Inhibitors vs. Scramble-inhibitors, Figure 2B).

Cell length, width, and the ratio of cellular length to cellular width (cell aspect ratio) were analyzed with ImageJ. As shown in Figure 2C, up-regulation of let-7a-5p led to not much change in cell length but significantly increased cell width, resulting in significantly decreased cell aspect ratio (4.3 ± 0.5 vs. 3.5 ± 0.3, Scramble-mimics vs. Mimics, *p* < 0.01), whereas down-regulation of let-7a-5p led to increased cell length and decreased cell width, resulting in significantly increased cell aspect ratio (5.8 ± 0.3 vs. 11.2 ± 0.8, Scramble-inhibitors vs. Inhibitors, *p* < 0.01). These data indicate that let-7a-5p modulates cell morphology by regulating the distribution of stress fibers.

### Let-7a-5p Modulates Cell Stiffness and Traction Force of ASMCs

**FIGURE 3:**
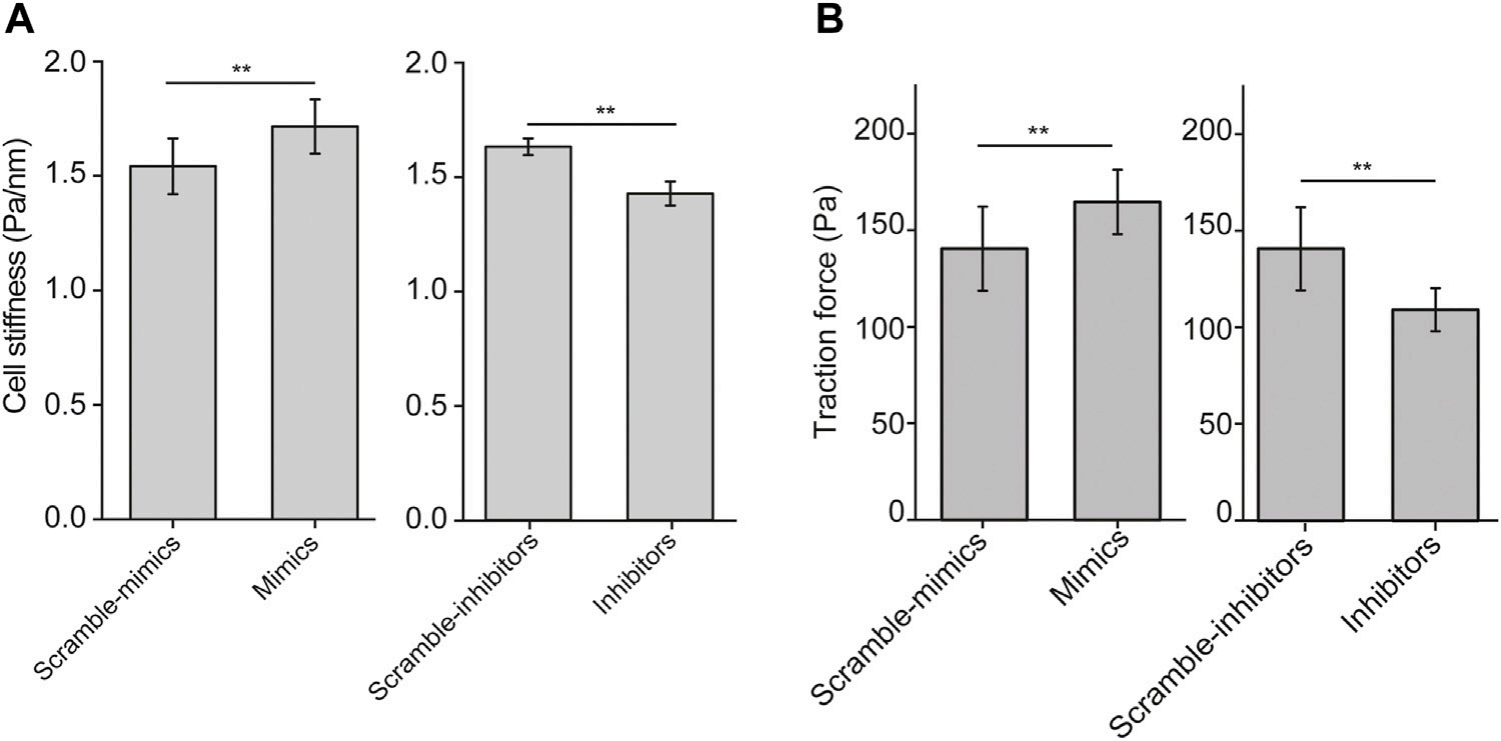
Cell stiffness and traction force generated by ASMCs transfected with let-7a-5p mimics, inhibitors and scramble controls. (A) Cell stiffness of ASMCs transfected with let-7a-5p mimics, inhibitors and scramble controls measured by OMTC (100 Hz). (B) Quantified traction forces of the cells transfected with let-7a-5p mimics, inhibitors and scramble controls. Data are means ± S.E.M. n = 50–100 cells for **(A)** and 10-20 cells for **(B)**. (*p < 0.05, **p < 0.01).

Since cell aspect ratio and the distribution of stress fibers influence biomechanical behaviors of the cell, we then examined cell stiffness and traction force of ASMCs after transiently transfected with let-7a-5p mimics, inhibitors or corresponding controls (Scramble-mimics, and Scramble-inhibitors) for 48 h by OMTC and traction force assay, respectively. We found that let-7a-5p up-regulation significantly increased cell stiffness (1.54 ± 0.12 vs. 1.71 ± 0.11, Scramble-mimics vs. Mimics, *p* < 0.01), whereas let-7a-5p down-regulation significantly decreased cell stiffness (1.60 ± 0.04 vs. 1.41 ± 0.05, Scramble-inhibitors vs. Inhibitors, *p* < 0.01) (Figure 3A).

Traction force in a single cell measured by observing the cell contraction-induced displacement of fluorescent beads embedded in the underneath elastic substrate exhibited distinct pattern of force distribution when the cell was treated in different conditions (Supplementary Figure S3). Quantitatively as shown in Figure 3B, let-7a-5p up-regulation significantly increased traction force (140 ± 21 vs. 164 ± 16, Scramble-mimics vs. Mimics, *p* < 0.01), whereas let-7a-5p down-regulation significantly decreased traction force (138 ± 17 vs. 112 ± 16, Scramble-inhibitors vs. Inhibitors, *p* < 0.01). These data indicate that let-7a-5p indeed modulates the biomechanical behaviors of ASMCs.

Furthermore, we transfected ASMCs with mimics, mimics + inhibitors, and scramble of let-7a-5p, respectively, and then measured the stiffness and traction force of these cells. The data indicate that let-7a-5p inhibitor rescued the effect of let-7a-5p mimics on cell stiffness and traction force, confirming that let-7a-5p indeed affects biomechanics of ASMCs (Supplementary Figure S4).

### Let-7a-5p Modulates Migration of ASMCs

**FIGURE 4:**
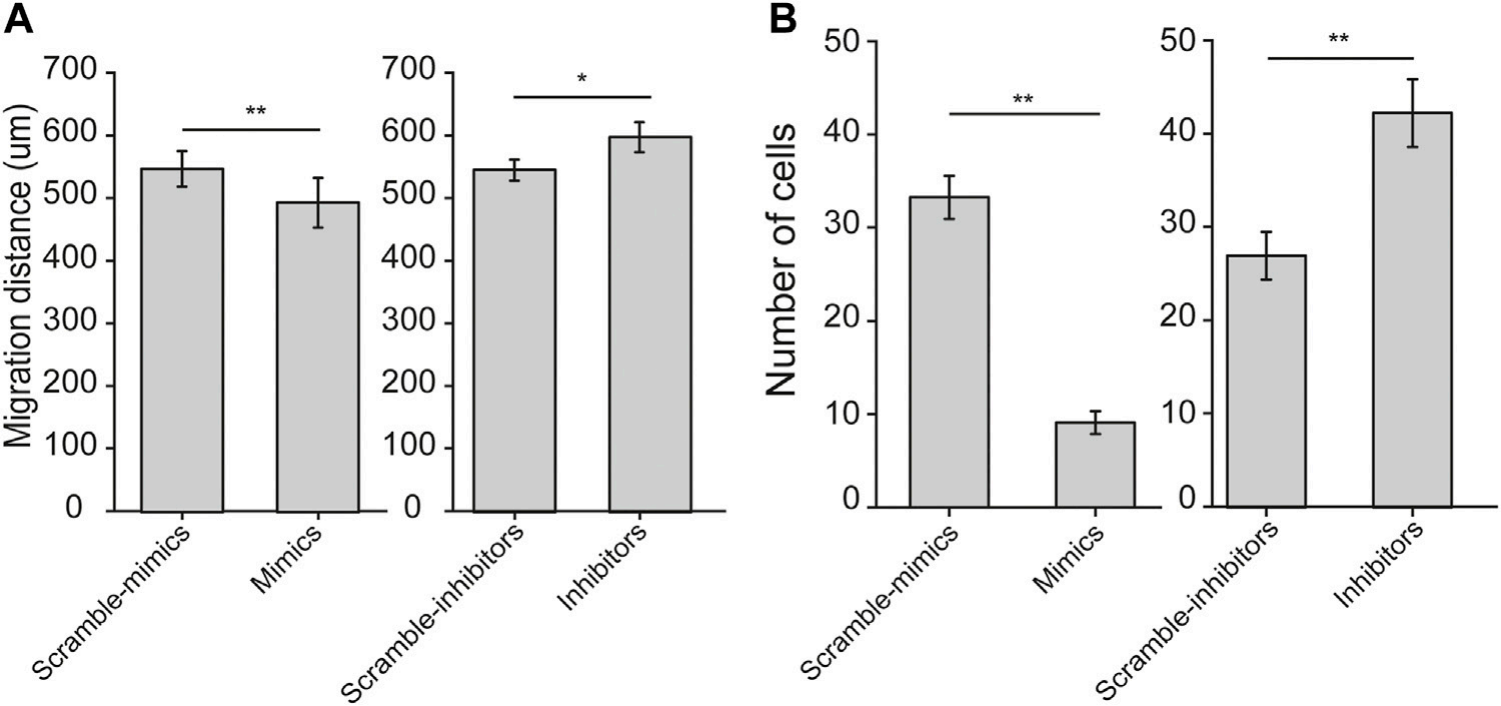
Horizontal and vertical cell migration of ASMCs transiently transfected with let-7a-5p mimics, inhibitors, and related scramble controls measured by wound-healing and transwell assays, respectively. **(A)** Bar plot shows the migration distance of ASMCs at 24 h to show horizontal migration analyzed in wound healing assays. **(B)** Bar plot shows the number of migrated cells to show vertical migration analyzed in transwell assays. Data are means ± S.E.M., n = 3. *p < 0.05, **p < 0.01.

Since both cell stiffness and traction force are determinants of cell migration, we thus examined the effects of let-7a-5p on migration of ASMCs, in terms of either horizontal migration by wound healing assay or vertical migration by transwell assay. We found that let-7a-5p up-regulation or down-regulation seemed to inhibit or enhance horizontal cell migration over 24 h, respectively (Supplementary Figure S5). The quantitative results of migration distance of ASMCs at 24 h indicate indeed that let-7a-5p up-regulation significantly inhibited horizontal cell migration, whereas let-7a-5p down-regulation significantly promoted horizontal cell migration (559 ± 19 vs. 493 ± 21 for Scramble-mimics vs. Mimics, and 548 ± 16 vs. 598 ± 23 μm, for Scramble-inhibitors vs. Inhibitors, respectively, *p* < 0.01, Figure 4A).

Similarly, let-7a-5p up-regulation or down-regulation appeared to inhibit or enhance vertical cell migration as shown in the representative images of transwell assay (Supplementary Figure S6). The quantitative results show that let-7a-5p up-regulation decreased, whereas let-7a-5p down-regulation increased the number of ASMCs migrated to the bottom well of transwell chamber in 24 h, both significantly (33 ± 2 vs. 9 ± 1, for Scramble-mimics vs. Mimics, 27 ± 3 vs. 43 ± 4 for Scramble-inhibitors vs. Inhibitors, respectively, *p* < 0.01, Figure 4B). Together, these results suggest that let-7a-5p negatively modulates migration of ASMCs.

### Let-7a-5p Modulates Mechanotransduction-Associated mRNAs of ASMCs

**FIGURE 5:**
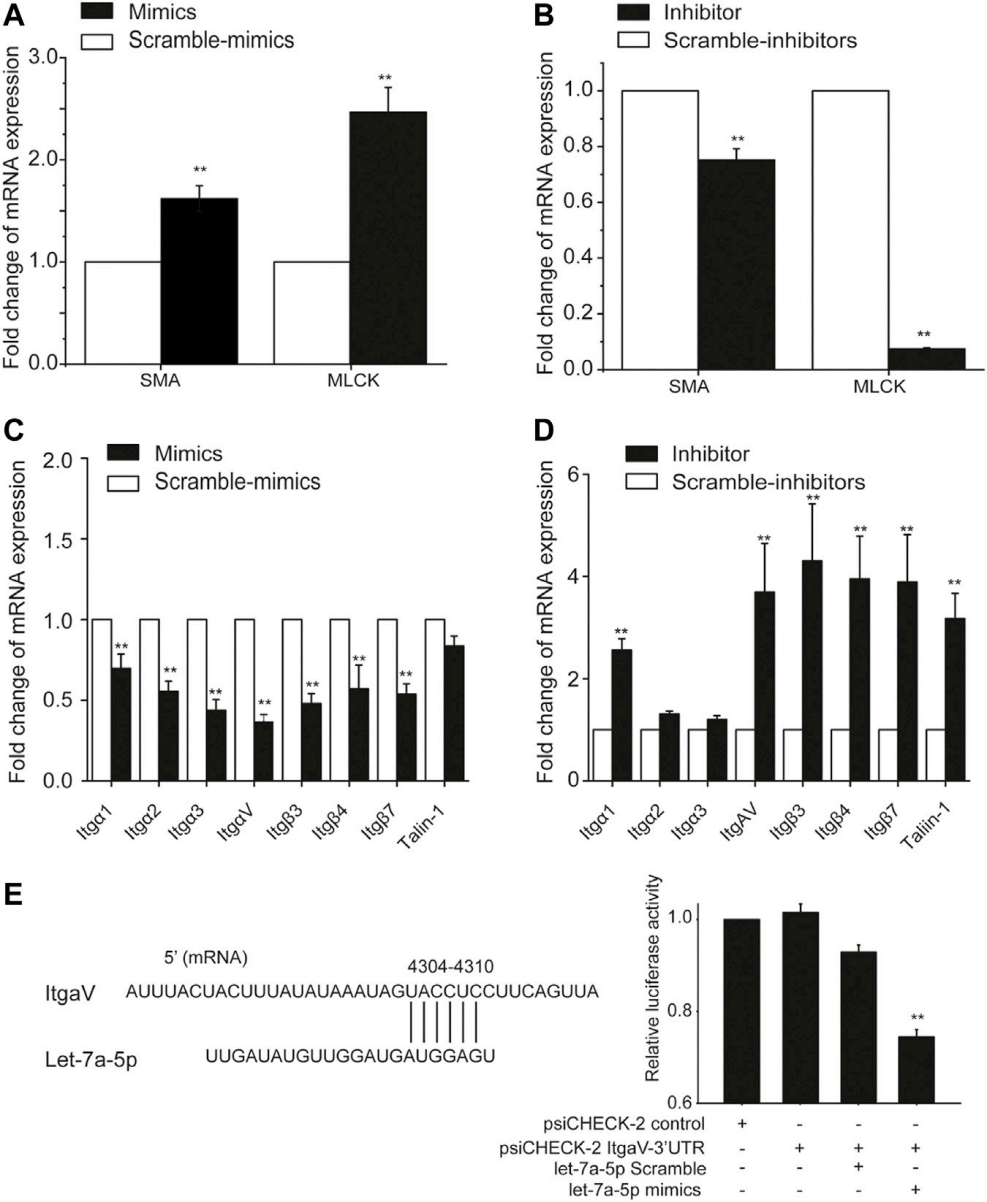
The expression of mRNA associated with biomechanical properties in ASMCs detected with mRNA qRT-PCR. The fold change of mRNA expression of MLCK and SMA in ASMCs after treatment with let-7a-5p mimics **(A)**, inhibitors **(B)**, and related scramble controls. The fold change of mRNA expression of focal adhesion-associated molecules such as integrin, vinculin, and talin in ASMCs after treatment with let-7a-5p mimics **(C)**, inhibitors **(D)**, and related scramble controls. **(E)** ItgαV was targeted by let-7a-5p analyzed by double-luciferase experiments. The schematic sequence cloned in psiCHECK plasmids was shown on left, and the relative luciferase activity in HEK293T cells treated with or without let-7a-5p mimics was shown in right. Data are means ± S.E.M., n = 3. **p < 0.01.

Since cell stiffness and traction force are regulated by *α*-smooth muscle actin (SMA) and myosin light chain kinase (MLCK), we therefore measured the mRNA expression of SMA and MLCK in ASMCs with mRNA qRT-PCR after treatment with let-7a-5p mimics, inhibitors, and corresponding scramble controls. The data show that compared to scramble control groups, let-7a-5p up-regulation enhanced mRNA expression of SMA by ∼1.7 fold (*p* < 0.01) and MLCK by ∼2.5 fold (*p* < 0.01) (Figure 5A), whereas let-7a-5p down-regulation inhibited that of SMA by ∼0.7 fold (*p* < 0.01) and MLCK by ∼0.07 fold (*p* < 0.01) (Figure 5B).

In addition to these contraction/relaxation-associated molecules, we also evaluated the effects of let-7a-5p on the mRNA expression profile of molecules associated with focal adhesion in ASMCs such as Itgα1/2/3/4/5/6/7/8/10/11/V/X, Itgβ1/3/4/5/7, Talin1/2 and Vinculin, because not only they are essential in the regulation of cell structure and function but some of them have also been reported to be directly targeted by this miRNA (Müller and Bosserhoff, 2008). The data show that mRNA expression level of these molecules in ASMCs ranked in the order of Itgβ1 > ItgαV > Vinculin > Itgα4 > Talin1 > Itgβ5 > Itgα1 > Itgα6 > Itgα2 > Itgα3 > Itgα8 > Talin2 > Itgβ4 > Itgα7 > Itgβ3 > Itgβ7 > ItgαX > Itgα10 > Itgα11 > Itgα5 (Supplementary Figure S7). Furthermore, in ASMCs let-7a-5p up-regulation clearly inhibited the mRNA expression of Itgα1, Itgα2, Itgα3, ItgαV, Itgβ3, Itgβ4, Itgβ7, and Talin1 (Mimics vs. Scramble-mimics, *p* < 0.01, Figure 5C) whereas let-7a-5p down-regulation enhanced the mRNA expression of these molecules except Itgα2 and Itgα3 (Inhibitors vs. Scramble-inhibitors, *p* < 0.01, Figure 5D), These results suggest that let-7a-5p may modulate the structure and function of ASMCs through multiple mechanotransduction signaling pathways.

Considering the changes of mRNA expression of these integrins, we further conducted an extensive search of potential common target molecules of let-7a-5p in the miRmap database. To further explore the biological function of the target genes of let-7a-5p, Go enrichment and KEGG pathway analysis were performed using DAVID database. GO analysis results showed that mechanotransduction structure and process of cells such as filopodium, lamellipodium, acting binding, and cell migration have been mainly targeted by let-7a-5p (Supplementary Figure S8A). In addition, KEGG pathway analyses indicated that target genes of let-7a-5p were significantly enriched in signaling pathways of PI3K-Akt, MAPK and AMPK, and in ECM-receptor interaction, focal adhesion, amoebiasis, which are all involved in the process of mechanotransduction (Supplementary Figure S8B).

Interestingly, several integrins such as Itgα1, Itgα2, ItgαV, Itgβ3, and Itgβ7 have been predicted to be targeted by let-7a-5p. Among them, ItgαV in the ASMCs appeared to be the most abundant in expression (Supplementary Figure S7) and most responsive to high stretch (Figure 5C). We thus explored the extent of direct cross-talk between let-7a-5p and ItgαV by using dual-luciferase assays in classical model cell line HEK293T. As shown in Figure 5E, intracellular transfection of the psiCHECK-2 ItgαV-3′UTR construct did not significantly decrease the luciferase production in HEK293T cells as compared to their counterparts transfected with the psiCHECK-2 control vector. In contrast, the cells transfected with both psiCHECK-2 ItgαV-3′UTR construct and let-7a-5p mimics showed a 26% reduction in luciferase production as compared to those transfected with psiCHECK-2 ItgαV-3′UTR construct and let-7a-5p Scramble, indicating occurrence of actual binding between let-7a-5p and ItgαV. These data suggest that let-7a-5p may indeed directly target integrins and interfere with their expression in ASMCs.

## Discussion

In this study, we mainly revealed in ASMCs *in vitro* that among the 29 miRNAs associated with airway inflammation, let-7a-5p was most responsive to MV-simulating high stretch (13%) with expression increased by nearly 10-fold as compared to others by less than 4-fold, suggesting that let-7a-5p may be the predominating stretch-responsive miRNA. Following that, we revealed that up-regulation of let-7a-5p increased cell stiffness and traction force as well as SMA/MLCK expression but inhibited cell migration both horizontally and vertically. In contrast, down-regulation of let-7a-5p resulted in opposite effects on ASMCs. These findings demonstrate that miRNA let-7a-5p in ASMCs could respond with a great extent of increase in expression to high stretch that is comparable to that imposed on airways by MV. Consequently, the up-regulation of let-7a-5p could lead to modulation of ASMCs both structurally and functionally, particularly the morphology and biomechanics of the cells. These findings provide not only an alternative mechanism responsible for high stretch-induced effects on mechanobiology of ASMCs, but also potentially a novel therapeutic target for high stretch-related lung pathology such as VILI.

As a potentially life-threatening complication, VILI has been attributed to cellular response to excessive stress (barotrauma) or strain (volutrauma) applied to the lung parenchyma, or shear stress occurring at the interface of open and closed lung regions (atelectrauma), in addition to cellular response to inflammatory factors (biotrauma) (Tremblay et al., 1997; Pinhu et al., 2003; Uhlig and Uhlig, 2011; Chen et al., 2018). Compared to other cell types such as epithelial cells and macrophages that are widely studied for their roles in VILI, ASMCs have been paid little attention to their participation in these processes even though they are widely known as the major mechanosensitive components in airways. There are two reports that ASMCs respond to stretch stimulation by changing their morphology and contractile phenotype, which is attributable to the protective role of deep inspiration in healthy subjects and loss of such protection in asthmatics (Bossé et al., 2013; Wright et al., 2013). In this study, we found that up-regulation of let-7a-5p led to increased cell stiffness and traction force as well as enhanced expression of contractile phenotype markers including SMA and MLCK whereas down-regulation of let-7a-5p led to opposite effects, suggesting that let-7a-5p is a contractile phenotype promotor of ASMCs. Since high stretch simulating the strain induced by MV increased the expression of let-7a-5p in ASMCs, our experimental results indicate that *in vivo* continuing high stretch during MV may well stimulate ASMCs to increase the expression level of stretch-responsive miRNAs such as let-7a-5p, which in turn promotes stiffening and force generation of ASMCs.

Such alteration in stiffness and force generation of ASMCs may contribute to decreased lung compliance and corresponding deterioration of VILI. Firstly, stiffened ASMCs would increase lung parenchyma stress in both radial and axial directions and thus stiffen the alveoli by interdependence forces, which makes the whole lung “stiffer” or less compliant (Mitzner et al., 1992; Shapiro and Bartlett, 1992). Secondly, enhanced force generation of ASMCs would lead to greater narrowing of airways, which makes the lung “smaller” (Cortes-Puentes et al., 2015). Consequently, delivery of a pre-determined tidal volume in the volume-controlled model in MV would require a greater airway pressure, which is likely to over distend alveoli and ultimately lead to barotrauma and volutrauma, respectively (Gattinoni et al., 2017). Consistent with these findings, a recent study reports that late-stage COVID-19 patients with ARDS often show low lung compliance (Pan et al., 2020), and thus lowering tidal volume for MV and bronchial dilator are recommended for treatment of these patients in order to both avoid overdistension of alveoli and improve the lung compliance (Wright et al., 1994; Mauri et al., 2017; Farooqi et al., 2020; Guérin et al., 2022).

In this study, we also revealed that up or down-regulation of let-7a-5p decreased or increased cellular length and cell aspect ratio, respectively. Interestingly, the morphology of ASMCs with let-7a-5p down-regulation was very similar to that of freshly isolated ASMCs (Deng et al., 2006), suggesting that let-7a-5p may have a critical role in regulating the morphogenesis of ASMCs. And such worm-like structures of ASMCs are thought to be conducive to forming synchronous winding muscle fibers to facilitate force generation and transmission *in vivo*, Furthermore, morphology and mechanics of animal cells are known to be regulated by focal adhesion which is a kind of membrane-spanning structure with components such as integrin, vinculin and talin (Kechagia et al., 2019). These molecules connect extracellular matrix and intracellular cytoskeletal system so that to mediate basic cellular functions such as adhesion, growth and migration (Ehrig et al., 2019). In this study, we tested the expression of some of these molecules, integrin and talin in particular in ASMCs with/out regulation of let-7a-5p. We found that up or down-regulation of let-7a-5p led to inhibition or promotion of mRNA expression of subfamily members of integrin and talin, namely, Itgα1, Itgα2, Itgα3, ItgαV, Itgβ3, Itgβ4, Itgβ7, and talin1, respectively. This suggests that let-7a-5p specifically interacts with these molecules to regulate ASMC morphology and mechanics.

As the most abundant miRNAs in the lung, let-7 family has been widely studied for their involvement in inflammatory diseases (Takamizawa et al., 2004; Polikepahad et al., 2010). And the subfamily member, let-7a-5p is known as a regulator of inflammatory response and cellular phenotype (Cho et al., 2015). It is generally considered that let-7a-5p mainly presents anti-inflammatory properties through repression of specific genes targeting downstream signaling pathways such as Smad2 and STAT3 and thus repressed TGF-β2-induced cell migration, invasion, and epithelial-mesenchymal transformation (Cheng et al., 2017; Hu et al., 2017; Chen et al., 2019; Liu and Jiang, 2020). Our results indicate that high stretch could significantly increase the expression of let-7a-5p in ASMCs. This seemed to suggest that stretch-induced high expression of let-7a-5p during MV might also play an anti-inflammatory role in lung pathology while adversely regulating stiffness and force generation of ASMCs to reduce lung compliance, a contradictory contribution to the underlying mechanism of VILI.

One limitation of this study is that we cannot confirm the primary mediator of let-7a-5p in regulating morphology and mechanics of ASMCs. In this study, we examined some of the key players in cytoskeleton dynamics and focal adhesion including SMA, MLCK, integrin and talin, and found that the mRNA expression of these molecules was associated with that of let-7a-5p as well as evidence of direct binding between let-7a-5p with ItgαV. The analysis by miRmap, however, did not find direct target of let-7a-5p on MLCK or SMA, which suggests that let-7a-5p may possibly regulate the expression of MLCK and SMA through targeting the transcription and degradation process of these molecules. As regards integrins and talin, they are known important signaling molecules for the formation of focal adhesion and related cytoskeletal structures which in most cases are positively correlated with cell stiffness in ASMCs despite some report of negative relationship between integrin expression and cell stiffness in tumor cells and airway epithelial cells (Chen et al., 2014; Chi et al., 2022). In addition to integrin and talin, there are many other cytoskeleton-correlated molecules including actin-related protein 2/3, calmodulin, casein kinase 2 interacting protein-1, diaphanous related formin 2, p21-protein activation kinase 1, and radixin, which have been identified as direct targets of let-7a-5p and may also involve in the response of ASMCs to high stretch (Safi et al., 2004; Müller and Bosserhoff, 2008; Hu et al., 2013; Tan et al., 2015; Tang et al., 2016; Zhou et al., 2017; Yang et al., 2018). We did not investigate these molecules in this study. We also did not investigate the specific regulatory mechanisms through which high stretch modulates the miRNA expression of let-7a-5p, even though a multitude of molecules including lncRNA H19, Lin28B, longab intergenic noncoding RNA COX2, and SYNCRIP have been reported to regulate the miRNA expression of let-7a (Li et al., 2009; Cheng et al., 2017; Hu et al., 2017; Chen et al., 2019; Sun et al., 2019; Brewster et al., 2020; Chen et al., 2020). Furthermore, other miRNAs that are abundant in the lung, especially other subfamily members of let-7a may also participate in regulation of morphology and mechanics of ASMCs (Polikepahad et al., 2010). All these need to be studied in future in order to fully understand the role and associated regulatory mechanisms of high stretch in lung homeostasis and injury.

## Conclusion

We revealed that high stretch was able to enhance the expression of miRNA let-7a-5p in ASMCs, which in turn led to alterations in morphology and mechanical behaviors of ASMCs in a way to reduce lung compliance. We also confirmed that regulation of let-7a-5p was associated with changing expression of cytoskeletal and focal adhesion molecules including SMA, MLCK and integrin together with evidence of direct binding between let-7a-5p and ItgαV, a subfamily member of integrin. These findings not only provide an alternative perspective to improve our understanding of mechanical behaviors and their regulatory pathway of ASMCs in response to high stretch, but also suggest that miRNAs such as let-7a-5p may be important biomarkers to facilitate diagnosis and therapeutic targets for preventing/treating high stretch-related lung disease.

## Data Availability

The raw data supporting the conclusions of this article will be made available by the authors, without undue reservation.

## Abbreviations

ANOVA, analysis of variance; ARDS, acute respiratory distress syndrome; ASMCs, airway smooth muscle cells; BP, biological process; BSA, bovine serum albumin; CC, cellular component; CCK-8, cell counting Kit-8; COVID-19, coronavirus disease 2019; CT, cycle threshold; DAVID, Database for Annotation, Visualization, and Integrated Discovery; DMEM, Dulbecco’s modified Eagle’s medium; FBS, fetal bovine serum; GO, Gene Ontology; HMDD, human MicroRNA disease database; IL-13, interleukin 13; Itg, integrin; KEGG, Kyoto Encyclopedia of Genes and Genomes; MF, molecular function; miRNAs, microRNAs; MLCK, myosin light chain kinase; MV, mechanical ventilation; OMTC, optical magnetic twisting cytometry; PBS, phosphate buffered saline; qRT-PCR, quantitative reverse transcription PCR; RT, room temperature; SMA, α-smooth muscle actin; 2D, two dimensional; VILI, ventilator-induced lung injury.
