# Supplementary material for: MircroRNA Let-7a-5p in Airway Smooth Muscle Cells is Most Responsive to High Stretch in Association With Cell Mechanics Modulation
Source: Front Physiol. 2022 Mar 25;13:830406. doi: 10.3389/fphys.2022.830406 (PMC8990250; doi:10.3389/fphys.2022.830406)
Supplement: Supplementary file 3 [file Table1.DOCX]

Table S1: Overview of miRNA correlated with chronic respiratory diseases (1, 2)

| No. | miRNA | Function | References |
| --- | --- | --- | --- |
| 1 | let-7a-5p | Let-7a was indicated to inhibit the proliferation and promote the apoptosis of human ASMCs, which may be associated with the downregulation of STAT3 expression | (3) |
| 2 | let-7b-5p | Antagonism of let-7b did not significantly inhibit Th2-cell cytokine production or mucus production, eosinophil infiltrates, or AHR | (4) |
| 3 | let-7b-3p | Let-7b-3p inhibits tumor growth and metastasis by targeting the BRF2-mediated MAPK/ERK pathway in human lung adenocarcinoma | (5) |
| 4* | let-7c-3p | Let-7c-3p regulates autophagy under oxidative stress by targeting ATG3 in Lens Epithelial cells | (6) |
| 5 | let-7d-5p | Lung cancer cell-derived exosomal let-7d-5p down-regulates OPRM1 to promote cancer-induced bone Pain | (7) |
| 6 | let-7e-5p | In AR, levels of Th2 cytokines were upregulated in the nasal mucosa of currently symptomatic subjects, whereas let7e was downregulated in currently nonsymptomatic AR | (8) |
| 7* | let-7e-3p |  |  |
| 8 | let-7f-5p | Hypoxia-induced let-7f-5p/TARBP2 feedback loop regulates osteosarcoma cell proliferation and invasion by inhibiting the Wnt signaling pathway | (9) |
| 9* | let-7f-1-3p | This causes increased cellular expression of let-7f, which then binds to the 39UTR of ADRB2 and represses ADRB2 translation, leading to a progressive loss of β_2_AR protein expression. | (10) |
| 10* | let-7f-2-3p | IL-17A is associated with severe asthma and requires IL-23 receptor signaling, which is negatively regulated by let-7f microRNA | (11) |
| 11* | let-7i-5p | Extracellular vesicles-encapsulated let-7i shed from bone mesenchymal stem cells suppress lung cancer via KDM3A/DCLK1/FXYD3 axis | (12) |
| 12 | miR-10a-3p | PDGF-BB promotes the cell proliferation of ASMCs through miR-10a/BDNF. Knock-down of GAS5 significantly decreased airway hyperresponsiveness in asthmatic rats | (13) |
| 13 | miR-18a-3p | MiR-18a deficiency enhanced CCR6, RORγt, Th17 cell differentiation in vitro and increased the number of tissue Th17 cells expressing CCR6, RORγt and IL-17A in airway inflammation models in vivo | (14) |
| 14* | miR-19a-5p | Low miR-19a expression in ASMCs from asthmatic patients is the key event that results in constitutive increased PRMT1 expression and remodeling. | (15) |
| 15 | miR-19a-3p | MiR-19a-3p may serve as a tumor suppressor partly through the regulation of UBAP2L expression in NSCLC | (16) |
| 16 | miR-21-3p | MiR-21 overexpression significantly reduced the expression of PTEN, while PTEN knock-down markedly increased HASM cell proliferation and migration | (17) |
| 17 | miR-27a-5p | Mesenchymal stromal/stem cells modulate response to experimental sepsis-induced lung injury via regulation of miR-27a-5p in recipient mice | (18) |
| 18 | miR-27a-3p | MiR.27a.3p targets multiple proteins of intracellular signaling networks that regulate the activity of nuclear factor kB and mitogen activated protein kinases | (19) |
| 19* | miR-126-3p | Selective blockade of microRNA-126 suppressed the asthmatic phenotype, resulting in diminished T(H)2 responses, inflammation, airways hyperresponsiveness, eosinophil recruitment, and mucus hypersecretion | (20) |
| 20* | miR-128-1-5p | MIR155HG contributed to the apoptosis and inflammation of HPMECs in smoke-related COPD by regulating miR-128-5p/BRD4 axis | (21) |
| 21 | miR-146b-3p | MiR-146a and miR-146b can negatively regulate COX-2 and IL-1 expression at pharmacological levels | (22) |
| 22* | miR-155-3p | MiR-155 may enhance COX-2 expression in hASMCs by promoting PI3K/AKT inactivation of butyrate response factor 1 and K homology splicing regulatory protein, thereby enhancing COX-2 mRNA stability | (23) |
| 23* | miR-221-3p | Leads to a diminished anti-inflammatory response and drives M2-macrophages to exhibit a M1-cytokine profile | (24) |
| 24* | miR-221-5p | MicroRNA-221 regulates the hyperproliferation of airway smooth muscle cells and the release of IL-6 from patients with severe asthma | (25) |
| 25 | miR-449a | MiR-449a regulates autophagy to inhibit silica-induced pulmonary fibrosis through targeting Bcl2 | (26) |
| 26 | miR-449c-5p | MiR-449 is essential in regulating airway ciliated cells by targeting NOTCH1 | (27) |
| 27* | miR-570-3p | MiR570-3p upregulated numerous cytokines and chemokines (CCL4, CCL5, TNFα, and IL-6) and also enhanced their induction by TNFα | (28) |
| 28* | MiR-629-5p | MiR-629-5p promotes the invasion of lung adenocarcinoma via increasing both tumor cell invasion and endothelial cell permeability | (29) |
| 29 | miR-629-3p | Transfecting human bronchial epithelial cells with miR-629-3p mimic induced epithelial IL-8 mRNA and protein expression. IL-1b and IL-8 protein levels were significantly increased in sputum of patients with severe asthma | (30) |

Note: * means that the Ct of this miRNA > 36

References

1. Sastre B, Canas J A, Rodrigo-Munoz J M, Del Pozo V. Novel Modulators of Asthma and Allergy: Exosomes and MicroRNAs. *Frontiers in immunology*. (2017) 8:826.doi:10.3389/fimmu.2017.00826

2. Chen J, Hu C, Pan P. Extracellular Vesicle MicroRNA Transfer in Lung Diseases. *Frontiers in physiology*. (2017) 8:1028.doi:10.3389/fphys.2017.01028

3. Chen Y, Qiao L, Zhang Z, Hu G, Zhang J, Li H. Let-7a inhibits proliferation and promotes apoptosis of human asthmatic airway smooth muscle cells. *Exp Ther Med*. (2019) 17:3327-3334.doi:10.3892/etm.2019.7363

4. Collison A, Mattes J, Plank M, Foster P S. Inhibition of house dust mite-induced allergic airways disease by antagonism of microRNA-145 is comparable to glucocorticoid treatment. *The Journal of allergy and clinical immunology*. (2011) 128:160-167 e4.doi:10.1016/j.jaci.2011.04.005

5. Li Y, Dong R, Lu M, Cheng C, Feng Z, Zhao R, et al. Let-7b-3p inhibits tumor growth and metastasis by targeting the BRF2-mediated MAPK/ERK pathway in human lung adenocarcinoma. *Transl Lung Cancer Res*. (2021) 10:1841-1856.doi:10.21037/tlcr-21-299

6. Li T, Huang Y, Zhou W, Yan Q. Let-7c-3p Regulates Autophagy under Oxidative Stress by Targeting ATG3 in Lens Epithelial Cells. *Biomed Res Int*. (2020) 2020:6069390.doi:10.1155/2020/6069390

7. Li X, Chen Y, Wang J, Jiang C, Huang Y. Lung Cancer Cell-Derived Exosomal let-7d-5p Down-Regulates OPRM1 to Promote Cancer-Induced Bone Pain. *Front Cell Dev Biol*. (2021) 9:666857.doi:10.3389/fcell.2021.666857

8. Suojalehto H, Toskala E, Kilpelainen M, Majuri M L, Mitts C, Lindstrom I, et al. MicroRNA profiles in nasal mucosa of patients with allergic and nonallergic rhinitis and asthma. *Int Forum Allergy Rhinol*. (2013) 3:612-20.doi:10.1002/alr.21179

9. Chen G, Gu H, Fang T, Zhou K, Xu J, Yin X. Hypoxia-induced let-7f-5p/TARBP2 feedback loop regulates osteosarcoma cell proliferation and invasion by inhibiting the Wnt signaling pathway. *Aging (Albany NY)*. (2020) 12:6891-6903.doi:10.18632/aging.103049

10. Kim D, Cho S, Woo J A, Liggett S B. A CREB-mediated increase in miRNA let-7f during prolonged beta-agonist exposure: a novel mechanism of beta2-adrenergic receptor down-regulation in airway smooth muscle. *FASEB J*. (2018) 32:3680-3688.doi:10.1096/fj.201701278R

11. Newcomb D C, Cephus J Y, Boswell M G, Fahrenholz J M, Langley E W, Feldman A S, et al. Estrogen and progesterone decrease let-7f microRNA expression and increase IL-23/IL-23 receptor signaling and IL-17A production in patients with severe asthma. *The Journal of allergy and clinical immunology*. (2015) 136:1025-34 e11.doi:10.1016/j.jaci.2015.05.046

12. Liu J, Feng Y, Zeng X, He M, Gong Y, Liu Y. Extracellular vesicles-encapsulated let-7i shed from bone mesenchymal stem cells suppress lung cancer via KDM3A/DCLK1/FXYD3 axis. *J Cell Mol Med*. (2021) 25:1911-1926.doi:10.1111/jcmm.15866

13. Zhang X Y, Tang X Y, Li N, Zhao L M, Guo Y L, Li X S, et al. GAS5 promotes airway smooth muscle cell proliferation in asthma via controlling miR-10a/BDNF signaling pathway. *Life sciences*. (2018) 212:93-101.doi:10.1016/j.lfs.2018.09.002

14. Montoya M M, Maul J, Singh P B, Pua H H, Dahlstrom F, Wu N, et al. A Distinct Inhibitory Function for miR-18a in Th17 Cell Differentiation. *J Immunol*. (2017) 199:559-569.doi:10.4049/jimmunol.1700170

15. Sun Q, Liu L, Wang H, Mandal J, Khan P, Hostettler K E, et al. Constitutive high expression of protein arginine methyltransferase 1 in asthmatic airway smooth muscle cells is caused by reduced microRNA-19a expression and leads to enhanced remodeling. *The Journal of allergy and clinical immunology*. (2017) 140:510-524 e3.doi:10.1016/j.jaci.2016.11.013

16. Pan Y, Jin K, Xie X, Wang K, Zhang H. MicroRNA-19a-3p inhibits the cellular proliferation and invasion of non-small cell lung cancer by downregulating UBAP2L. *Exp Ther Med*. (2020) 20:2252-2261.doi:10.3892/etm.2020.8926

17. Liu Y, Yang K, Shi H, Xu J, Zhang D, Wu Y, et al. MiR-21 modulates human airway smooth muscle cell proliferation and migration in asthma through regulation of PTEN expression. *Experimental lung research*. (2015) 41:535-45.doi:10.3109/01902148.2015.1090501

18. Younes N, Zhou L, Amatullah H, Mei S H J, Herrero R, Lorente J A, et al. Mesenchymal stromal/stem cells modulate response to experimental sepsis-induced lung injury via regulation of miR-27a-5p in recipient mice. *Thorax*. (2020) 75:556-567.doi:10.1136/thoraxjnl-2019-213561

19. Regev K, Paul A, Healy B, von Glenn F, Diaz-Cruz C, Gholipour T, et al. Comprehensive evaluation of serum microRNAs as biomarkers in multiple sclerosis. *Neurol Neuroimmunol Neuroinflamm*. (2016) 3:e267.doi:10.1212/NXI.0000000000000267

20. Mattes J, Collison A, Plank M, Phipps S, Foster P S. Antagonism of microRNA-126 suppresses the effector function of TH2 cells and the development of allergic airways disease. *Proc Natl Acad Sci U S A*. (2009) 106:18704-9.doi:10.1073/pnas.0905063106

21. Song J, Wang Q, Zong L. LncRNA MIR155HG contributes to smoke-related chronic obstructive pulmonary disease by targeting miR-128-5p/BRD4 axis. *Biosci Rep*. (2020) 40:10.1042/BSR20192567

22. Comer B S, Camoretti-Mercado B, Kogut P C, Halayko A J, Solway J, Gerthoffer W T. MicroRNA-146a and microRNA-146b expression and anti-inflammatory function in human airway smooth muscle. *American journal of physiology. Lung cellular and molecular physiology*. (2014) 307:L727-34.doi:10.1152/ajplung.00174.2014

23. Comer B S, Camoretti-Mercado B, Kogut P C, Halayko A J, Solway J, Gerthoffer W T. Cyclooxygenase-2 and microRNA-155 expression are elevated in asthmatic airway smooth muscle cells. *American journal of respiratory cell and molecular biology*. (2015) 52:438-47.doi:10.1165/rcmb.2014-0129OC

24. Quero L, Tiaden A N, Hanser E, Roux J, Laski A, Hall J, et al. miR-221-3p Drives the Shift of M2-Macrophages to a Pro-Inflammatory Function by Suppressing JAK3/STAT3 Activation. *Front Immunol*. (2019) 10:3087.doi:10.3389/fimmu.2019.03087

25. Perry M M, Baker J E, Gibeon D S, Adcock I M, Chung K F. Airway smooth muscle hyperproliferation is regulated by microRNA-221 in severe asthma. *Am J Respir Cell Mol Biol*. (2014) 50:7-17.doi:10.1165/rcmb.2013-0067OC

26. Han R, Ji X, Rong R, Li Y, Yao W, Yuan J, et al. MiR-449a regulates autophagy to inhibit silica-induced pulmonary fibrosis through targeting Bcl2. *J Mol Med (Berl)*. (2016) 94:1267-1279.doi:10.1007/s00109-016-1441-0

27. Taka S, Tzani-Tzanopoulou P, Wanstall H, Papadopoulos N G. MicroRNAs in Asthma and Respiratory Infections: Identifying Common Pathways. *Allergy Asthma Immunol Res*. (2020) 12:4-23.doi:10.4168/aair.2020.12.1.4

28. Roff A N, Craig T J, August A, Stellato C, Ishmael F T. MicroRNA-570-3p regulates HuR and cytokine expression in airway epithelial cells. *Am J Clin Exp Immunol*. (2014) 3:68-83.doi:

29. Li Y, Zhang H, Fan L, Mou J, Yin Y, Peng C, et al. MiR-629-5p promotes the invasion of lung adenocarcinoma via increasing both tumor cell invasion and endothelial cell permeability. *Oncogene*. (2020) 39:3473-3488.doi:10.1038/s41388-020-1228-1

30. Maes T, Cobos F A, Schleich F, Sorbello V, Henket M, De Preter K, et al. Asthma inflammatory phenotypes show differential microRNA expression in sputum. *The Journal of allergy and clinical immunology*. (2016) 137:1433-46.doi:10.1016/j.jaci.2016.02.018
