# Supplementary figures and images for: MircroRNA Let-7a-5p in Airway Smooth Muscle Cells is Most Responsive to High Stretch in Association With Cell Mechanics Modulation
Source: Front Physiol. 2022 Mar 25;13:830406. doi: 10.3389/fphys.2022.830406 (PMC8990250; doi:10.3389/fphys.2022.830406)

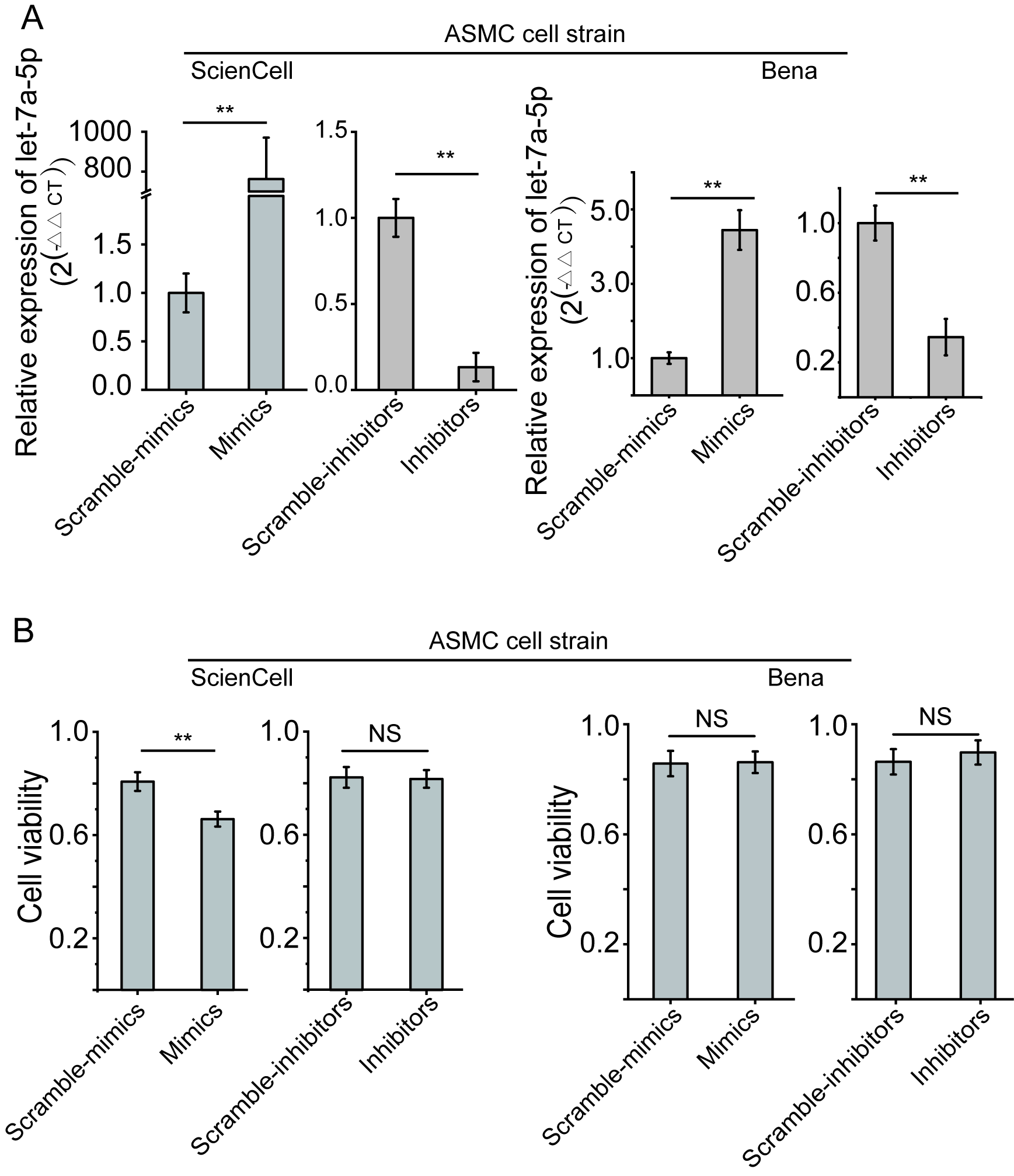

Supplement: Supplementary file 4 [file DataSheet1.zip › Image 1 (15).TIF]

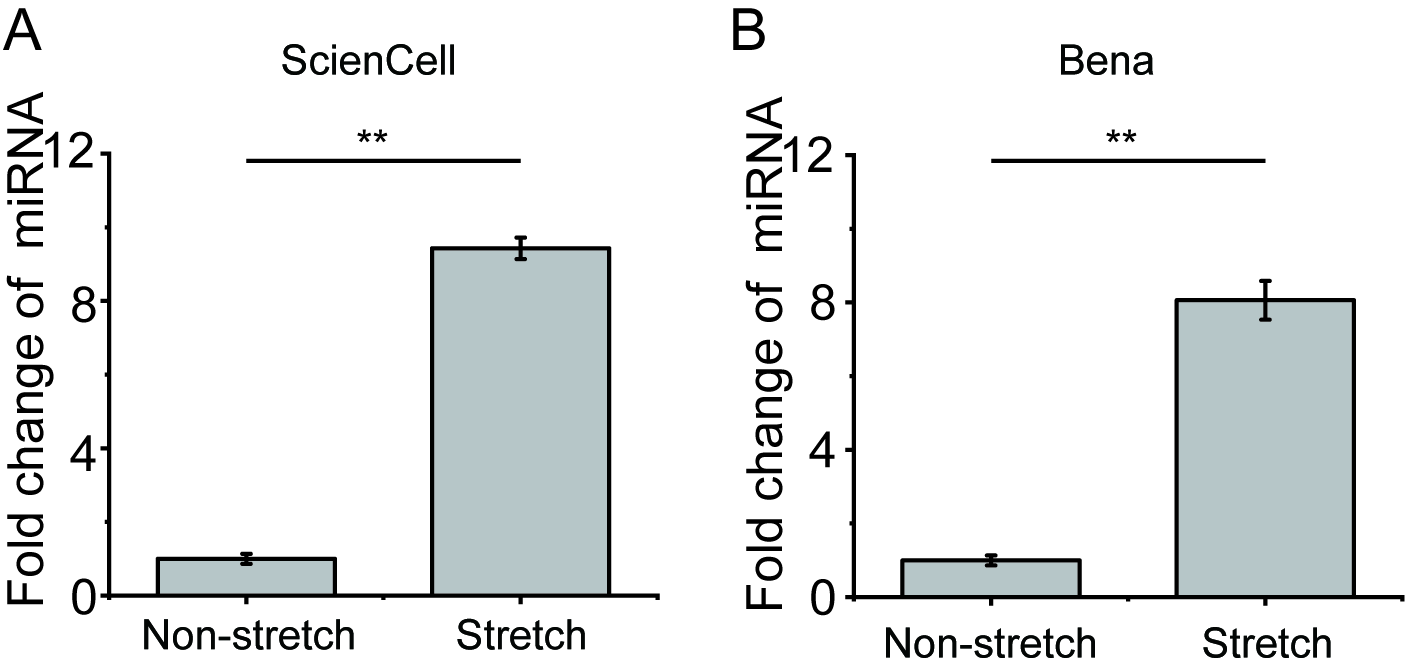

Supplement: Supplementary file 4 [file DataSheet1.zip › Image 2 (9).TIF]

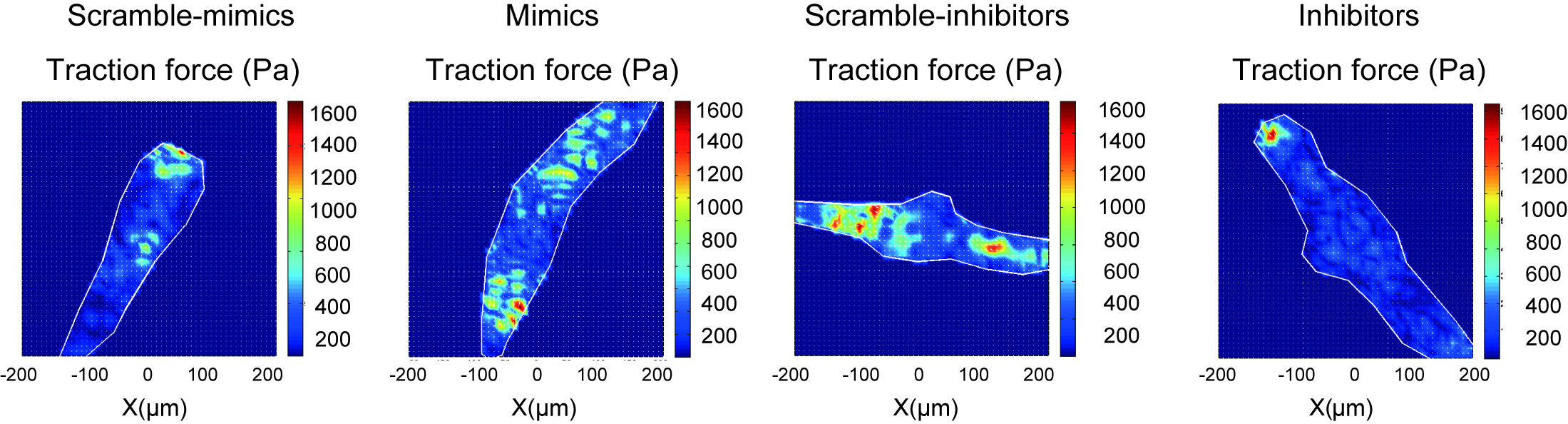

Supplement: Supplementary file 4 [file DataSheet1.zip › Image 3 (4).TIF]

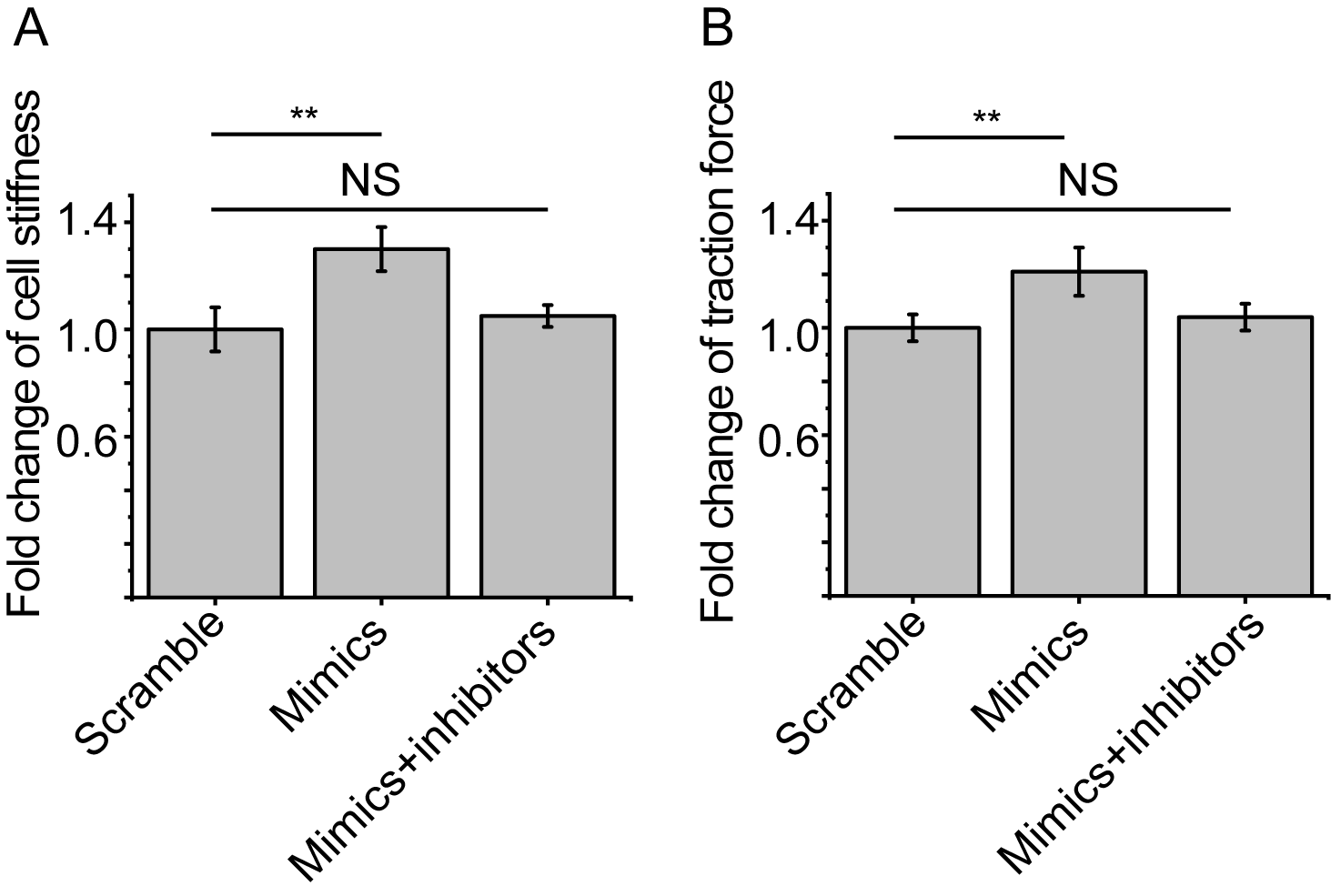

Supplement: Supplementary file 4 [file DataSheet1.zip › Image 4 (1).TIF]

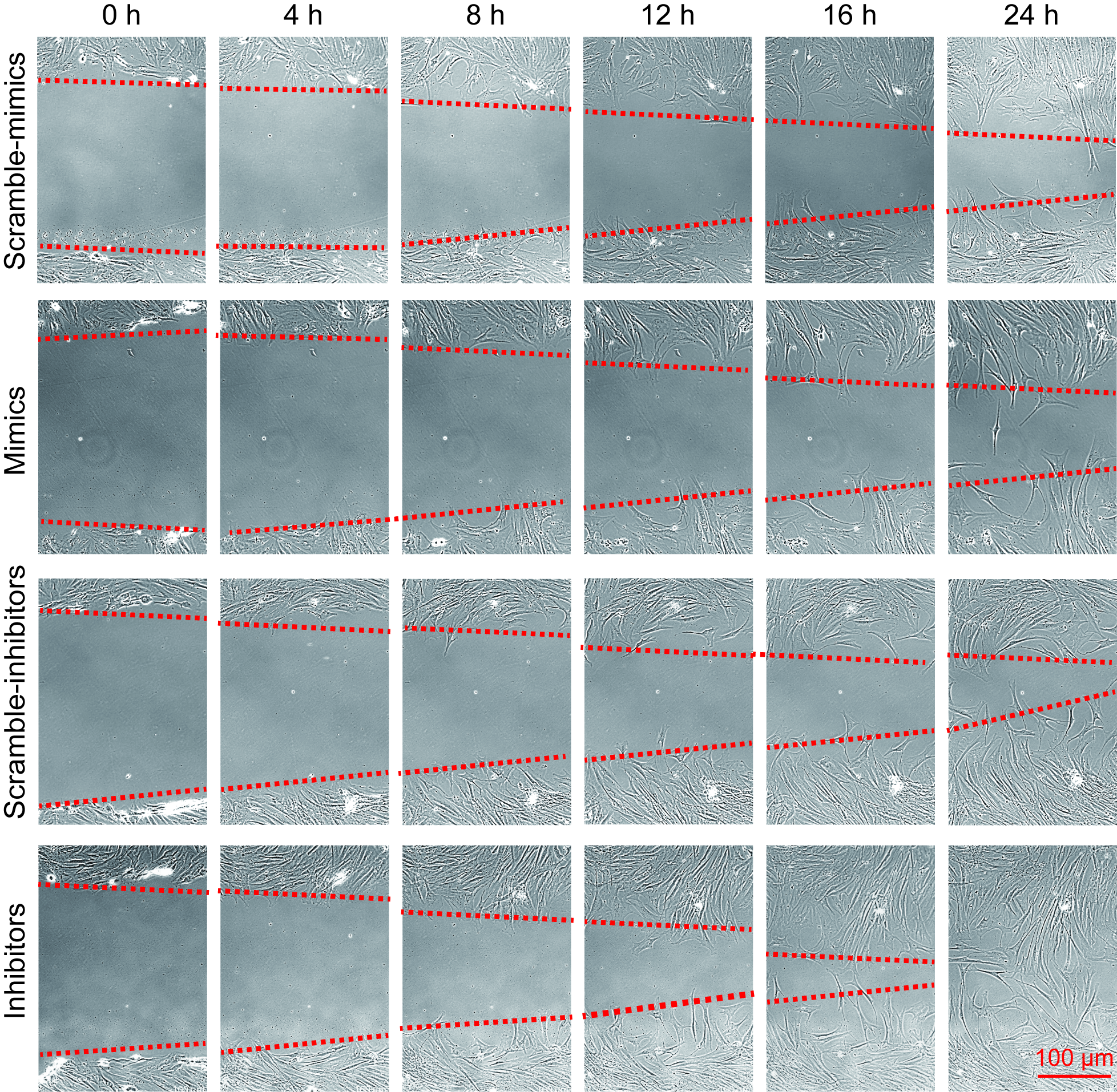

Supplement: Supplementary file 4 [file DataSheet1.zip › Image 5 (1).TIF]

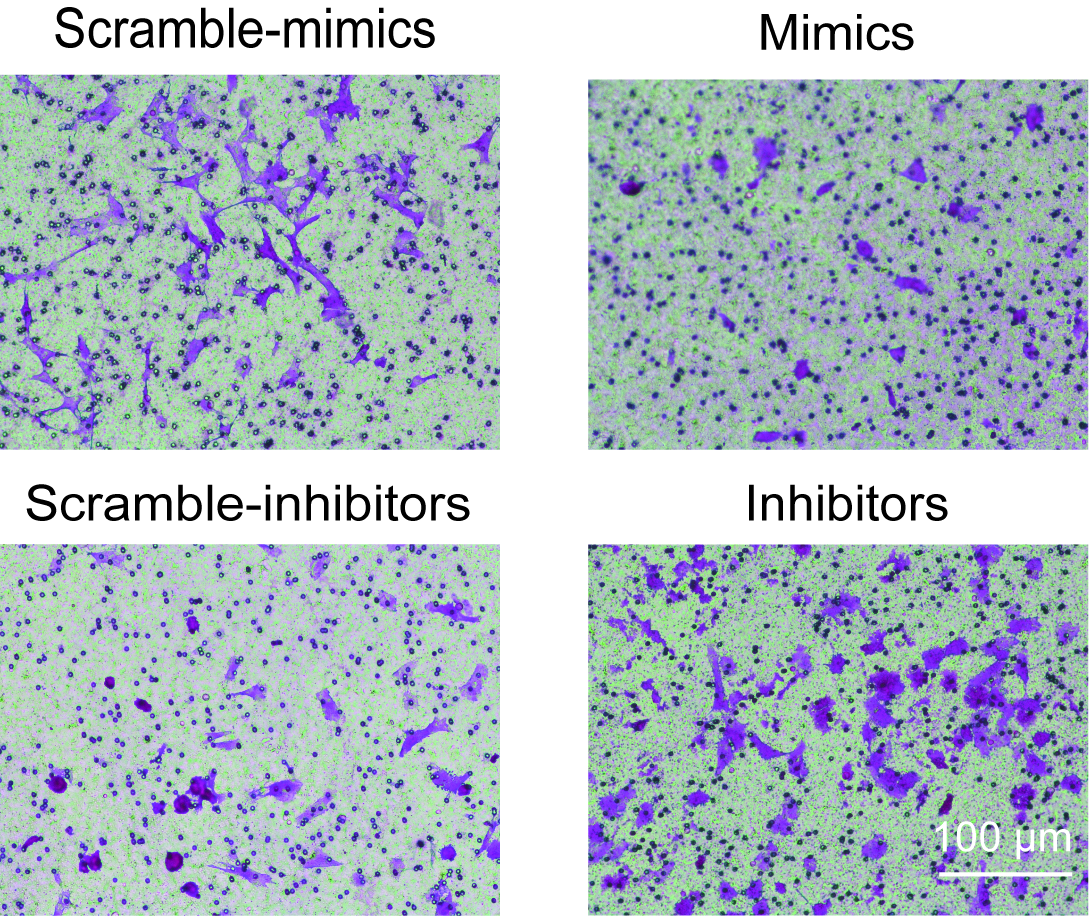

Supplement: Supplementary file 4 [file DataSheet1.zip › Image 6.TIF]

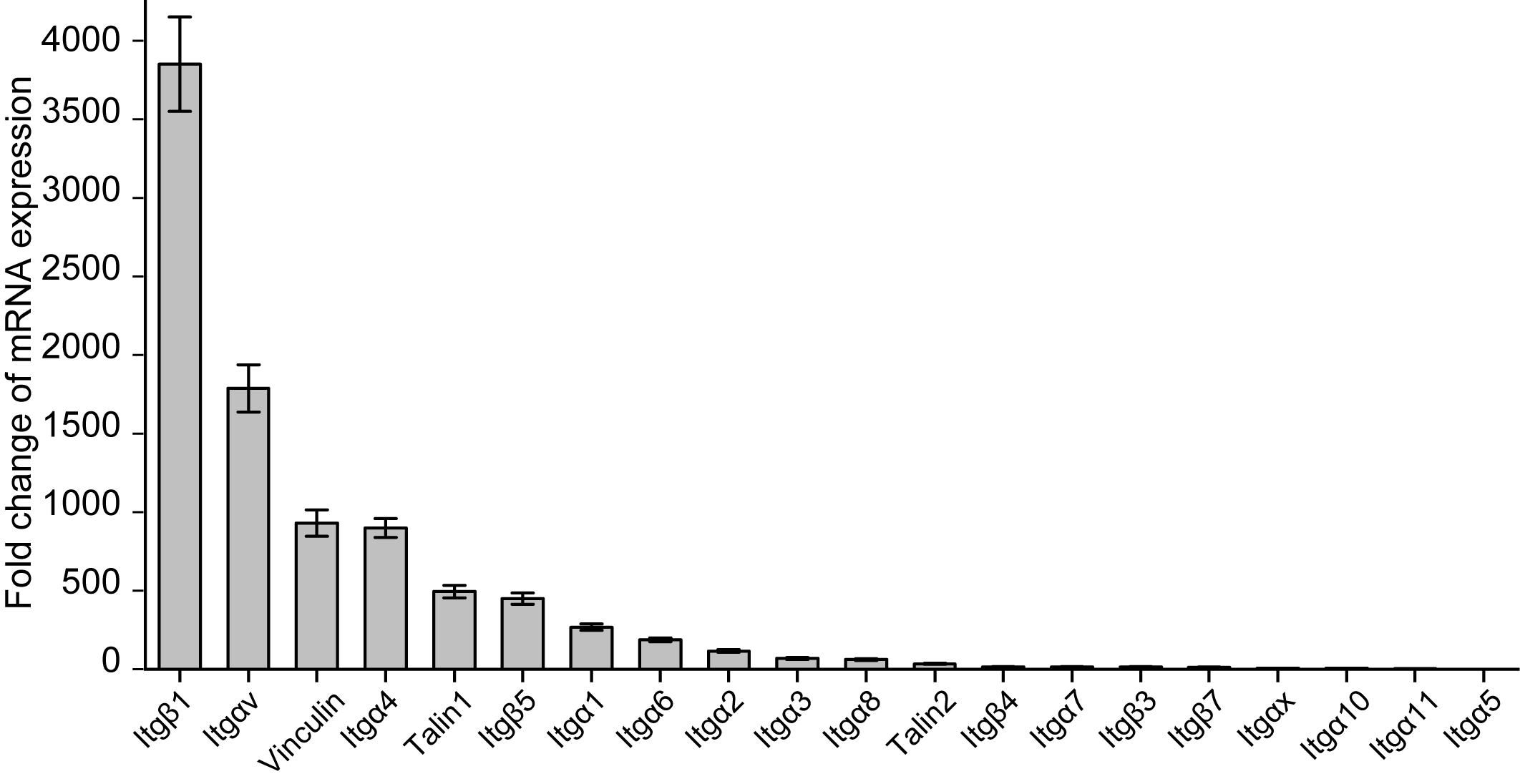

Supplement: Supplementary file 4 [file DataSheet1.zip › Image 7.TIF]

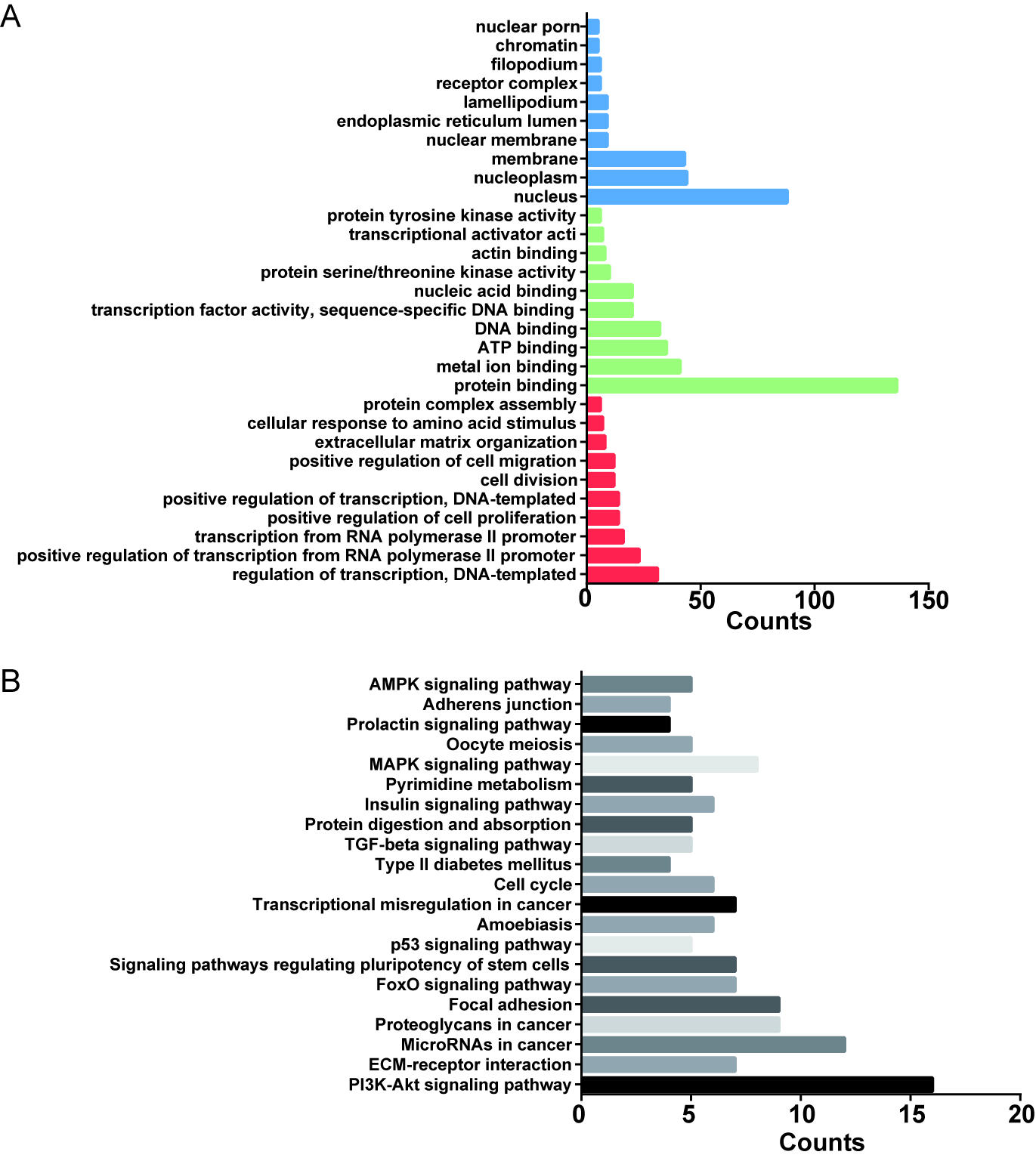

Supplement: Supplementary file 4 [file DataSheet1.zip › Image 8.TIF]
