# Supplementary material for: MircroRNA Let-7a-5p in Airway Smooth Muscle Cells is Most Responsive to High Stretch in Association With Cell Mechanics Modulation
Source: Front Physiol. 2022 Mar 25;13:830406. doi: 10.3389/fphys.2022.830406 (PMC8990250; doi:10.3389/fphys.2022.830406)
Supplement: Supplementary file 5 [file Table2.DOCX]

Table S2: Primer sequence of miRNA

| No. | MiRNA  Name | RT primer | Primer(forward) | Universal primer |
| --- | --- | --- | --- | --- |
| 1 | hsa-miR-221-3p | GTCGTATCCAGTGCAGGGTCCGAGGTATTCGCACTGGATACGACGAAACC | CGCGAGCTACATTGTCTGCTG | AGTGCAGGGTCCGAGGTATT |
| 2 | hsa-miR-221-5p | GTCGTATCCAGTGCAGGGTCCGAGGTATTCGCACTGGATACGACAAATCT | CGCGACCTGGCATACAATGT | AGTGCAGGGTCCGAGGTATT |
| 3 | hsa-miR-10a-3p | GTCGTATCCAGTGCAGGGTCCGAGGTATTCGCACTGGATACGACTATTCC | CGCGCAAATTCGTATCTAGG | AGTGCAGGGTCCGAGGTATT |
| 4 | hsa-miR-146b-3p | GTCGTATCCAGTGCAGGGTCCGAGGTATTCGCACTGGATACGACACCAGA | CGGCCCTGTGGACTCAGT | AGTGCAGGGTCCGAGGTATT |
| 5 | hsa-miR-570-3p | GTCGTATCCAGTGCAGGGTCCGAGGTATTCGCACTGGATACGACGCAAAG | CGCGCGAAAACAGCAATTAC | AGTGCAGGGTCCGAGGTATT |
| 6 | hsa-miR-18a-3p | GTCGTATCCAGTGCAGGGTCCGAGGTATTCGCACTGGATACGACCCAGAA | CGACTGCCCTAAGTGCTCC | AGTGCAGGGTCCGAGGTATT |
| 7 | hsa-miR-27a-5p | GTCGTATCCAGTGCAGGGTCCGAGGTATTCGCACTGGATACGACTGCTCA | GCGAGGGCTTAGCTGCTTG | AGTGCAGGGTCCGAGGTATT |
| 8 | hsa-miR-27a-3p | GTCGTATCCAGTGCAGGGTCCGAGGTATTCGCACTGGATACGACGCGGAA | GCGCGTTCACAGTGGCTAAG | AGTGCAGGGTCCGAGGTATT |
| 9 | hsa-miR-128-1-5p | GTCGTATCCAGTGCAGGGTCCGAGGTATTCGCACTGGATACGACTCTCAG | CGGGGCCGTAGCACTGT | AGTGCAGGGTCCGAGGTATT |
| 10 | hsa-miR-155-3p | GTCGTATCCAGTGCAGGGTCCGAGGTATTCGCACTGGATACGACTGTTAA | GCGCGCTCCTACATATTAGCA | AGTGCAGGGTCCGAGGTATT |
| 11 | hsa-miR-449a | GTCGTATCCAGTGCAGGGTCCGAGGTATTCGCACTGGATACGACACCAGC | CGCGTGGCAGTGTATTGTTA | AGTGCAGGGTCCGAGGTATT |
| 12 | hsa-miR-449c-5p | GTCGTATCCAGTGCAGGGTCCGAGGTATTCGCACTGGATACGACACAGCC | GCGTAGGCAGTGTATTGCTAGC | AGTGCAGGGTCCGAGGTATT |
| 13 | hsa-miR-629-5p | GTCGTATCCAGTGCAGGGTCCGAGGTATTCGCACTGGATACGACAGTTCT | GCGTGGGTTTACGTTGGG | AGTGCAGGGTCCGAGGTATT |
| 14 | hsa-miR-629-3p | GTCGTATCCAGTGCAGGGTCCGAGGTATTCGCACTGGATACGACGCTGGG | GCGGTTCTCCCAACGTAAG | AGTGCAGGGTCCGAGGTATT |
| 15 | hsa-miR-19a-5p | GTCGTATCCAGTGCAGGGTCCGAGGTATTCGCACTGGATACGACTGTAGT | CGCGAGTTTTGCATAGTTGC | AGTGCAGGGTCCGAGGTATT |
| 16 | hsa-miR-19a-3p | GTCGTATCCAGTGCAGGGTCCGAGGTATTCGCACTGGATACGACTCAGTT | GCGTGTGCAAATCTATGCAA | AGTGCAGGGTCCGAGGTATT |
| 17 | hsa-miR-126-3p | GTCGTATCCAGTGCAGGGTCCGAGGTATTCGCACTGGATACGACCGCATT | CGCGTCGTACCGTGAGTAAT | AGTGCAGGGTCCGAGGTATT |
| 18 | hsa-let-7b-5p | GTCGTATCCAGTGCAGGGTCCGAGGTATTCGCACTGGATACGACAACCAC | GCGCGTGAGGTAGTAGGTTGT | AGTGCAGGGTCCGAGGTATT |
| 19 | hsa-let-7b-3p | GTCGTATCCAGTGCAGGGTCCGAGGTATTCGCACTGGATACGACGGGAAG | GCGCGCTATACAACCTACTGC | AGTGCAGGGTCCGAGGTATT |
| 20 | hsa-let-7e-3p | GTCGTATCCAGTGCAGGGTCCGAGGTATTCGCACTGGATACGACGGAAAG | CGCGCTATACGGCCTCCTAG | AGTGCAGGGTCCGAGGTATT |
| 21 | hsa-let-7f-1-3p | GTCGTATCCAGTGCAGGGTCCGAGGTATTCGCACTGGATACGACGGGAAG | GCGCGCTATACAATCTATTGC | AGTGCAGGGTCCGAGGTATT |
| 22 | hsa-let-7f-2-3p | GTCGTATCCAGTGCAGGGTCCGAGGTATTCGCACTGGATACGACGGAAAG | CGCGCGCTATACAGTCTACTGT | AGTGCAGGGTCCGAGGTATT |
| 23 | hsa-let-7i-5p | GTCGTATCCAGTGCAGGGTCCGAGGTATTCGCACTGGATACGACAACAGC | CGCGCGTGAGGTAGTAGTTTGT | AGTGCAGGGTCCGAGGTATT |
| 24 | hsa-let-7c-3p | GTCGTATCCAGTGCAGGGTCCGAGGTATTCGCACTGGATACGACGGAAAG | GCGCGCTGTACAACCTTCTAG | AGTGCAGGGTCCGAGGTATT |
| 25 | hsa-let-7a-5p | GTCGTATCCAGTGCAGGGTCCGAGGTATTCGCACTGGATACGACAACTAT | GCGCGTGAGGTAGTAGGTTGT | AGTGCAGGGTCCGAGGTATT |
| 26 | hsa-let-7d-5p | GTCGTATCCAGTGCAGGGTCCGAGGTATTCGCACTGGATACGACAACTAT | GCGCGAGAGGTAGTAGGTTGC | AGTGCAGGGTCCGAGGTATT |
| 27 | hsa-let-7e-5p | GTCGTATCCAGTGCAGGGTCCGAGGTATTCGCACTGGATACGACAACTAT | CGCGTGAGGTAGGAGGTTGT | AGTGCAGGGTCCGAGGTATT |
| 28 | hsa-let-7f-5p | GTCGTATCCAGTGCAGGGTCCGAGGTATTCGCACTGGATACGACAACTAT | CGCGCGTGAGGTAGTAGATTGT | AGTGCAGGGTCCGAGGTATT |
| 29 | hsa-miR-21-3p | GTCGTATCCAGTGCAGGGTCCGAGGTATTCGCACTGGATACGACACAGCC | GCGCAACACCAGTCGATG | AGTGCAGGGTCCGAGGTATT |
