# Supplementary material for: MircroRNA Let-7a-5p in Airway Smooth Muscle Cells is Most Responsive to High Stretch in Association With Cell Mechanics Modulation
Source: Front Physiol. 2022 Mar 25;13:830406. doi: 10.3389/fphys.2022.830406 (PMC8990250; doi:10.3389/fphys.2022.830406)
Supplement: Supplementary file 7 [file Table3.DOCX]

Table S3: Primer sequence of miRNA

| No. | Gene Name | Primer (forward) | Primer (reverse) |
| --- | --- | --- | --- |
| 1 | MLCK | GATTTTGTGCTGCAGTGCTC | AGCGAGCGTACTGGATGG |
| 2 | SMA | CACAAGTATCACGGGAGAGC | TTGTCGTTCTTCACTGTTTTGG |
| 3 | Vinculin | CTCGTCCGGGTTGGAAAAGAG | AGTAAGGGTCTGACTGAAGCAT |
| 4 | Talin-1 | TACATGCTCCGAAATGGGGAC | ACCATGATCGTCTTCACAGTTC |
| 5 | Talin-2 | GTGAGTTTGGTGGATTTCAAGC | CTCCGCAGTTCTTATGCTCCT |
| 6 | ITGA1 | GCTCCTCACTGTTGTTCTACG | CGGGCCGCTGAAAGTCATT |
| 7 | ITGA2 | GGGAATCAGTATTACACAACGGG | CCACAACATCTATGAGGGAAGGG |
| 8 | ITGA3 | TCAACCTGGATACCCGATTCC | GCTCTGTCTGCCGATGGAG |
| 9 | ITGA4 | TCGGAGCCAGCATACTACC | CCACAGCACAGACAGAAGC |
| 10 | ITGA5 | ATTCACATCGCTCTCAAC | GTCTTCTCCACAGTCCAG |
| 11 | ITGA6 | CACATCTCCTCCCTGAGCAC | TATCTTGCCACCCATCCTTG |
| 12 | ITGA7 | GCTGTGAAGTCCCTGGAAGTGATT | GCATCTCGGAGCATCAAGTTCTT |
| 13 | ITGA8 | AGAATGGAGACCTTATTGTGGGA | GAGCCACTTCCGTCTGCTTT |
| 14 | ITGA9 | TCGTTCTTCGGCTACGCAG | CCCCATCCACTCATCATCGC |
| 15 | ITGA10 | CGAAGACTGGTAGGGAAACTGTTTA | GCCGACTGAGGTTCTTTGCT |
| 16 | ITGA11 | GACGGGAGACGTGTACAAGTGTC | CCGAGGCGCATGTTGTC |
| 17 | ITGAD | TCCCATCGTCCAACTGAAAGG | TGTACTTCTGCCCATCTGTGAT |
| 18 | ITGAE | TGCTGGCCGCTTTCAATGT | ACAGGATGGCAAAGGATTTCAT |
| 19 | ITGAL | CTTCTGTCCAGGGCATTAT | TGATGGTAGTGGCTGAGTTG |
| 20 | ITGAM | CTTAATACCATCGCATCCAAG | CTTCCTGTCTGAGTACCCTC |
| 21 | ITGAV | GACCCCTTACCCCAACTTTAT | TGACAGCCGAGACTGATTTTA |
| 22 | ITGA2B | CTTTGACCTCCGTGATGAGACC | CAGTCTTTTCTAGGACGTTCCAGTG |
| 23 | ITGAX | AGAGCTGTGATAAGCCAGTTCC | AATTCCTCGAAAGTGAAGTGTGT |
| 24 | ITGB1 | CCTACTTCTGCACGATGTGATG | CCTTTGCTACGGTTGGTTACATT |
| 25 | ITGB2 | CTCTCTCAGGAGTGCACGAA | CCCTGTGAAGTTCAGCTTCTG |
| 26 | ITGB3 | CATTACTCTGCCTCCACTACCA | AACGGATTTTCCCATAAGCA |
| 27 | ITGB4 | GCAGCTTCCAAATCACAGAGG | CCAGATCATCGGACATGGAGTT |
| 28 | ITGB5 | GAACGAGGCCAACGAGTACAC | CAAAGATGAGGTTGATGTTGTT |
| 29 | ITGB6 | TCCAGCTGATCATCTCAGCTTATG | TCATGTGAGAGCATTTCTTTTGGT |
| 30 | ITGB7 | AGAATGGCGGAATCCTCACCT | TGAAGTTCAGTTGCTTGCACC |
| 31 | ITGB8 | AATACTGTGAAAAGGATGACTTTTCTTGT | CCTTCCCAGCCCCTGAAG |
